# Prevalence of premenstrual syndrome, premenstrual dysphoric disorder, and dysmenorrhea in nursing students: a systematic review, meta-analysis, and evidence-based recommendations

**DOI:** 10.3389/fgwh.2025.1701704

**Published:** 2026-02-12

**Authors:** Sabyasachi Maity, Bharathi S. Gadad, Hansapani Rodrigo, Seham Noorani, Aneesha Usman, Chloe Lark, Mona Attarpour, Ivy Mageto, Lucas Schwartz, Anna Maria Trachuk, Dena Yaareb, Fadi Huzien, Nikhilesh Anand, Narendra Nayak, Jaime E. Mendoza, Shreya Nauhria, Samal Nauhria

**Affiliations:** 1 Department of Cellular and Integrative Physiology, Long School of Medicine, UT Health San Antonio, San Antonio, TX, United States; 2 Department of Medical Education, UTRGV School of Medicine, Edinburg, TX, United States; 3 School of Mathematical and Statistical Sciences, UTRGV, Edinburg, TX, United States; 4 Department of Medicine, Ross University School of Medicine, Bridgetown, Barbados; 5 Department of Medicine, University of Medicine and Health Sciences, Basseterre, Saint Kitts and Nevis; 6 Department of Medicine, Avalon University School of Medicine, Willemstad, Curaçao; 7 Department of Medicine, St. George’s University School of Medicine, True Blue, Grenada; 8 Department of Microbiology, St. Matthew’s University, Georgetown, Cayman Islands; 9 PrimeWest Consortium, San Dimas Community Hospital, Graduate Medical Education, San Dimas, CA, United States; 10 Child Protection, Cayman Islands Red Cross, Georgetown, Cayman Islands; 11 Civil Service College, Cayman Islands Government, Georgetown, Cayman Islands

**Keywords:** nursing students, menstrual disorders, premenstrual syndrome, dysmenorrhea, academic performance, wellbeing, premenstrual dysphoric disorder, women's health

## Abstract

**Introduction:**

Menstrual disorders—including premenstrual syndrome (PMS), premenstrual dysphoric disorder (PMDD), and dysmenorrhea—are highly prevalent among women of reproductive age and are associated with impaired academic performance, psychological distress, and reduced social functioning. Nursing students are particularly vulnerable due to the combined demands of intensive academic schedules and clinical training, yet prevalence estimates and institutional responses remain inconsistent.

**Methods:**

A systematic review and meta-analysis were conducted in accordance with PRISMA 2020 guidelines and registered in PROSPERO (CRD420251109363). PubMed, Scopus, Web of Science, CINAHL Ultimate, and APA PsycINFO were searched for studies published between 2016 and 2025. Eligible studies reported prevalence or impact of PMS, PMDD, or dysmenorrhea exclusively in nursing students. The Joanna Briggs Institute checklist for prevalence studies was used for quality appraisal. Random-effects meta-analysis was applied to calculate pooled prevalence estimates, and thematic synthesis was used to evaluate academic, social, and psychosocial impacts, coping strategies, and interventions.

**Results:**

Twenty studies involving 5,131 nursing students were included. The pooled prevalence was 62% for PMS, 21% for PMDD (including severe PMS), and 72% for dysmenorrhea, with substantial heterogeneity (*I*^2^ > 80%). Reported impacts included absenteeism, reduced concentration, diminished clinical performance, and impaired quality of life. Coping strategies were largely self-directed, including analgesics, rest, and dietary modifications, while few students accessed formal healthcare or institutional support. Only a limited number of studies evaluated structured interventions such as exercise, yoga, or nutritional supplementation.

**Conclusion:**

Menstrual disorders are highly prevalent among nursing students and carry significant academic, social, and psychological consequences. Nursing education programs should integrate routine screening, structured wellness initiatives, and evidence-based interventions to improve student wellbeing, reduce academic disruption, and strengthen workforce preparedness.

**Systematic Review Registration:**

https://www.crd.york.ac.uk/PROSPERO/view/CRD420251109363, PROSPERO CRD420251109363.

## 1 Introduction

Menstrual disturbances—including premenstrual syndrome (PMS), premenstrual dysphoric disorder (PMDD), and dysmenorrhea—are among the most prevalent and debilitating conditions affecting women of reproductive age worldwide (1, 2). Epidemiological studies estimate that up to 80% of women experience some form of premenstrual symptoms, with 30%–40% meeting criteria for clinically significant PMS and 3%–8% for PMDD, the most severe form (3, 4). Dysmenorrhea, or painful menstruation, affects an estimated 50%–90% of young women and is a leading cause of recurrent absenteeism from school and work, as well as diminished quality of life (5, 6).

Menstrual disorders are highly prevalent globally and significantly impair women's academic, occupational, and psychosocial functioning. Recent global analyses estimate that PMS affected 45.6% of women of childbearing age in 2021, with the number of cases nearly doubling since 1990 despite stable age-adjusted rates (7). Similarly, data from the Global Burden of Disease Study indicate a 46.5% increase in LMS cases, from 652.5 million in 1990 to 956.0 million in 2019, with the heaviest burden in low- and middle-income regions and among women aged 40–44 years (8). Dysmenorrhea remains one of the most common conditions, with systematic reviews reporting prevalence ranges from 60% to 73% among young women and up to 71% globally (9). These figures underscore the widespread and growing burden of menstrual disorders, highlighting the importance of population group-specific investigations, such as among nursing students.

Despite their ubiquity, these conditions are often under-recognized and trivialized, even though they profoundly affect academic achievement, social functioning, psychological wellbeing, and long-term career outcomes (5, 10). Untreated premenstrual disorders are associated with increased risk of anxiety, depression, substance misuse, and suicidality (1, 3). Reflecting this growing concern, the World Health Organization and national health agencies now frame menstrual health as a public health priority with implications for gender equity, workforce productivity, and sustainable health systems (2, 11).

Nursing students represent a particularly vulnerable subgroup within this landscape. Their intense academic and clinical schedules, coupled with the psychological stress of caregiving roles, may exacerbate menstrual symptoms and magnify their impact (12, 13). Studies consistently report that nursing students experience some of the highest global prevalence rates of PMS and dysmenorrhea among university populations, with consequences ranging from absenteeism and impaired concentration to reduced clinical performance and higher risk of mental health disorders (14–16). Importantly, their lived experiences not only affect their personal wellbeing but may also shape how they approach menstrual health in their future clinical practice (10, 17).

Our previous systematic review in medical students demonstrated similar findings, with pooled prevalence estimates of 51.3% for PMS, 17.7% for PMDD, and 72.7% for dysmenorrhea (18). The review also highlighted high levels of psychosomatic discomfort, self-medication practices, and significant academic and social disruption, underscoring the urgent need for institutional awareness and targeted interventions. While those findings advanced understanding in medical students, nursing students remain comparatively under-studied, despite their unique clinical responsibilities and frontline role in patient care.

To frame this complexity, the “Biopsychosocial Model” (19) provides a guiding lens, emphasizing how biological processes (hormonal fluctuations, prostaglandin release), psychological dimensions (stress, coping, mental health), and social factors (academic pressures, stigma, institutional support) jointly influence symptom severity and outcomes. The “Health Belief Model” (20) further clarifies how perceptions of susceptibility, perceived severity, and barriers to care affect the health-seeking behaviors and coping strategies of nursing students.

Against this backdrop, the current review aims to provide an up-to-date and methodologically rigorous synthesis of the prevalence and management strategies for PMS, PMDD, and dysmenorrhea in nursing students. By doing so, it addresses an important research gap and offers evidence-based recommendations for academic institutions, health policymakers, and curriculum designers to support menstrual health among this critical population.

The primary aims of the present systematic review and meta-analysis are as follows:

1. To estimate the pooled prevalence of PMS, PMDD, and dysmenorrhea among nursing students globally, prioritizing validated diagnostic criteria and measurement tools;
2. To synthesize evidence on the academic, social, and psychological consequences of menstrual disorders in nursing student populations;
3. To evaluate the effectiveness and feasibility of reported interventions and coping strategies; and
4. To identify key methodological gaps and provide recommendations for education policy, student support services, and future research.

Unaddressed menstrual health challenges have long-term consequences, contributing to burnout, absenteeism, and attrition from the nursing workforce. Creating a culture where menstrual health is validated as a legitimate wellness concern is therefore essential, both for gender equity and for workforce sustainability. By implementing reforms, nursing schools can model best practice for healthcare environments, signaling to students that menstrual health is not a private struggle but a core component of holistic patient care.

## 2 Methods

The systematic review and meta-analysis were conducted in accordance with the PRISMA 2020 (Preferred Reporting Items for Systematic Reviews and Meta-Analyses) statement (21) and guided by the Joanna Briggs Institute (JBI) Manual for Evidence Synthesis for prevalence and etiology reviews (22). The review protocol was registered prospectively in PROSPERO (CRD420251109363). As this study is a review of published literature, ethical approval was not required.

### 2.1 Search strategy

A comprehensive literature search was performed to identify eligible studies. The following databases were searched: PubMed, Scopus, Web of Science, CINAHL Ultimate, and APA PsycINFO (the latter two via EBSCOhost). Searches were conducted between 2016 and 2025 and restricted to English-language publications.

- PubMed: [“Students, Nursing” (Mesh)] AND “Premenstrual Syndrome” [Mesh],
- Scopus: TITLE-ABS-KEY(“nursing student*”) AND TITLE-ABS-KEY(“premenstrual*”),
- CINAHL Ultimate and APA PsycINFO (via EBSCOhost): TX nursing students AND (TX premenstrual syndrome OR TX premenstrual dysphoric disorder).

Additional searches for dysmenorrhea were conducted using comparable Boolean logic and subject headings in CINAHL Ultimate and APA PsycINFO. Supplementary searches included Google Scholar, citation snowballing, and manual screening of references from relevant reviews and included articles to ensure comprehensive coverage. Boolean operators (AND, OR), truncation (e.g., *), and database-specific subject headings (MeSH in PubMed, CINAHL Headings) were employed to maximize search sensitivity and specificity.

### 2.2 Definitions and MeSH headings

Searches used the MeSH term “Premenstrual Syndrome” (encompassing PMS and PMDD) and “Dysmenorrhea,” both of which are classified under the broader MeSH category of “Menstruation Disturbances.” PMDD is indexed as a subset of PMS in MeSH.

### 2.3 Study selection

All search results were exported to the EndNote reference management system. Duplicate records were removed prior to the screening process. Two reviewers (SN and SM) independently screened titles and abstracts for eligibility, followed by full-text review of potentially relevant articles. Disagreements were resolved through discussion or consultation with a third reviewer (ShN).

### 2.4 Eligibility criteria

Studies were eligible for inclusion if they reported prevalence data on premenstrual disorders—specifically PMS, PMDD, and/or dysmenorrhea—among populations consisting exclusively of nursing students. Only studies that provided clearly separated data for nursing students, with prevalence or quantitative outcomes distinctly reported, were included. Eligible designs comprised original quantitative studies such as cross-sectional, cohort, or prevalence studies. To ensure methodological rigor and comparability, only articles published in peer-reviewed English-language journals between 2016 and 2025 were considered.

**Figure 1:**
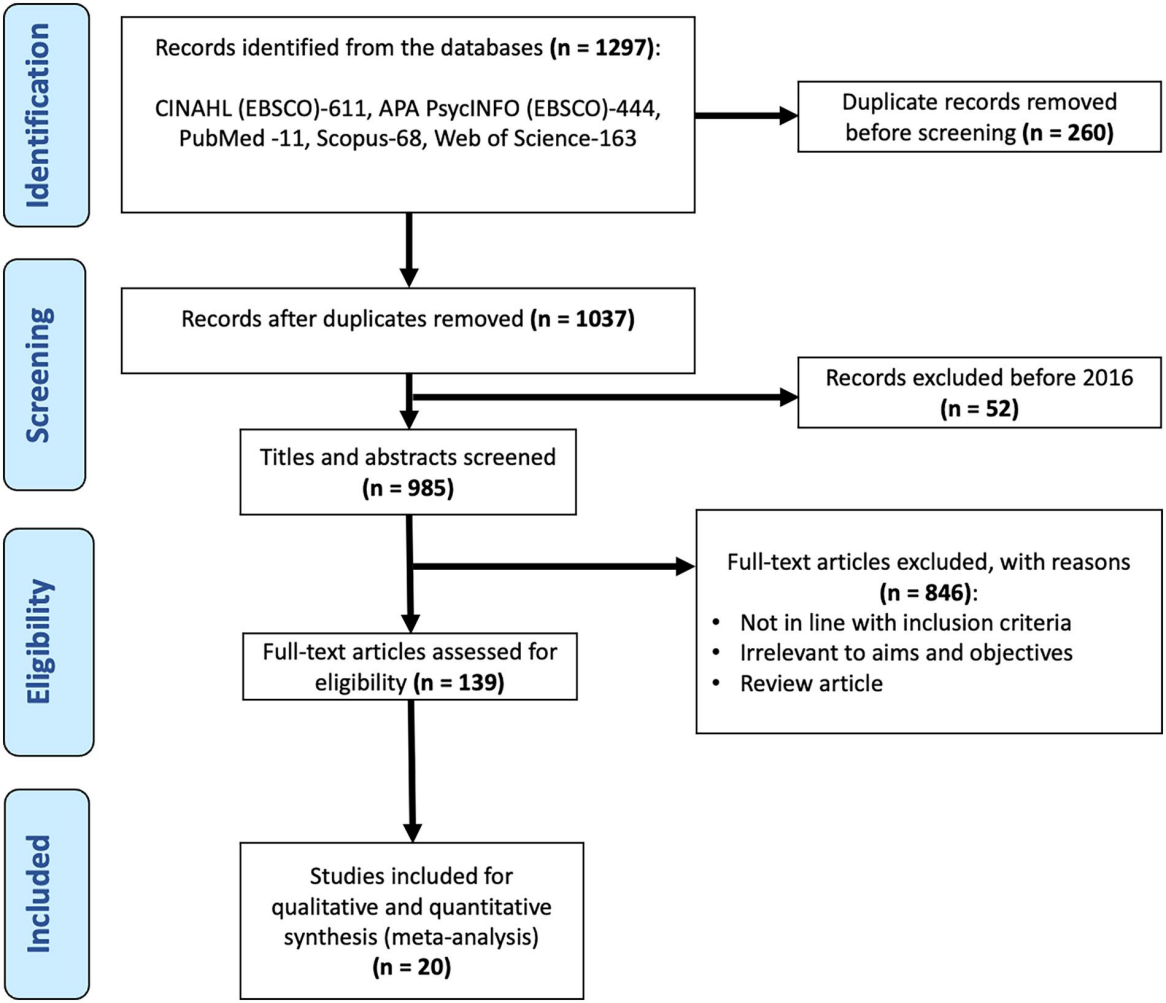
PRISMA 2020 flow diagram summarizing the study selection process.

Exclusion criteria were applied to maintain focus on the target population and ensure reliability of the findings. Studies that combined nursing students with other populations (e.g., medical or general university students) without providing separate data were excluded. Similarly, case reports, reviews, editorials, letters, qualitative-only studies, and conference abstracts without complete data were not eligible for inclusion. Finally, studies that did not report prevalence, outcomes, or relevant quantitative data on PMS, PMDD, or dysmenorrhea were excluded from the analysis. A PRISMA flow diagram is presented to illustrate the study selection process (Figure 1).

### 2.5 Data extraction and management

Ten reviewers, organized into five pairs, independently conducted data extraction. Each pair assessed titles, abstracts, and full texts of potentially eligible studies to verify adherence to inclusion criteria. Discrepancies within pairs were resolved through discussion, and if consensus was not reached, a third reviewer was consulted for arbitration. Extracted data included study details (author, year, country, sample size, and mean age), prevalence rates of PMS, PMDD, and dysmenorrhea, as well as information on academic and social impacts, interventions, and key conclusions. When data were missing or unclear, efforts were made to contact the study authors directly.

### 2.6 Risk of bias assessment

Methodological quality and risk of bias were assessed independently by two reviewers (JM, SN), using the JBI Critical Appraisal Checklist for Studies Reporting Prevalence Data. Any disagreements were resolved through discussion.

### 2.7 Data synthesis and analysis

#### 2.7.1 Quantitative synthesis

Where three or more studies reported prevalence of PMS, PMDD, or dysmenorrhea in nursing students, meta-analyses were conducted using a random-effects model (DerSimonian–Laird) with pooled prevalence estimates and 95% confidence intervals (CI). Given the clinical continuum from severe PMS to PMDD, and the DSM-5 conceptualization of PMDD as the most impairing end of the PMS spectrum (American Psychiatric Association, 2013), we pooled data from studies that reported combined PMDD/severe PMS prevalence. Statistical heterogeneity was assessed using the *I*^2^ statistic, with values greater than 75% considered substantial (23). Subgroup analyses were planned based on geographic region, measurement tool, or risk of bias, where sufficient data allowed. Sensitivity analyses were conducted by excluding studies at moderate or high risk of bias. Forest plots were generated to visualize individual study estimates and pooled prevalence under both common- and random-effects assumptions.

#### 2.7.2 Narrative and thematic synthesis

For outcomes not amenable to meta-analysis (e.g., academic/social impact, interventions), data were synthesized narratively and thematically. Thematic analysis was used to identify recurrent patterns in the academic and social consequences of menstrual disorders and in coping/intervention strategies. Six key themes were identified and are reported in the “Results.”

During data synthesis, the emergent themes regarding academic impact, psychosocial burden, coping strategies, and help-seeking behaviors were interpreted in consideration of the Biopsychosocial Model and the Health Belief Model. These frameworks helped structure our understanding of how biological, psychological, and sociocultural factors—as well as students' beliefs about their symptoms—influence the experience and management of premenstrual disorders among nursing students.

#### 2.7.3 Certainty of evidence

Certainty in the body of evidence for prevalence estimates was planned to be assessed using the GRADE approach, considering the following domains: risk of bias, inconsistency, indirectness, imprecision, and publication bias. Since most included studies were cross-sectional surveys, a formal quantitative GRADE rating was not possible. In such cases, a structured narrative appraisal of confidence in the findings was performed, highlighting key methodological strengths and limitations across the evidence base.

## 3 Results

### 3.1 Study selection

The initial database search yielded 1,037 unique records after automatic and manual duplicate removal. Following title and abstract screening, 985 article titles and abstracts were assessed, and a total of 52 articles were evaluated in full text. Of these, 20 studies met the inclusion criteria and were included in the systematic review and meta-analysis (Figure 1).

### 3.2 Study characteristics

The 20 included studies represented a cumulative sample of 5,131 nursing students across diverse geographical regions, including Asia, Europe, and the Middle East. The mean age of participants was 21.0 years (SD ≈ 1.3), ranging from 17 to 29 years. Sample sizes ranged from fewer than 100 participants to over 600 (Table 1).

**Table 1:** Study characteristics for all included studies.

Author (Year)	Place	Study design/data collection format	Data collection period	Population, age (years)	Reports on/scale used	Population studied (n)	PMS (%)	PMDD No. (%)	Dysmen (%)
Akalin and Karpuzluk (12)	Turkey	Descriptive and correlational, self-administered questionnaire (face-to-face/online)	Oct–Dec 2021	Mean age 20.3 ± 1.3 (18–25); menarche mean 13.2 ± 1.2 (10–18)	PMS Scale (PMSS); DASS	411	64.00%	Not reported	Not reported
Tekbaş and Güder (13)	N. Cyprus/Turkey (univ)	Descriptive, cross-sectional; online self-report survey	Dec 2020–Jan 2021	Mean age 21.1 ± 2.8 (18–26); menarche 13.2 ± 1.7	PMSS (Premenstrual Syndrome Scale)	322	74.0% moderate+; 33.5% severe	Not reported	75.50%
Qutishat et al. (24)	Oman	Descriptive correlational, cross-sectional, online self-report	Jan–Mar 2023	Female undergrads, 18–29 (mean not given)	PMSS; Attitude toward seeking psychological help	601	87.9% (62.1% low severity)	Not reported	Not reported
Lone and Singh (25)	Jammu, India	Cross-sectional, questionnaire-based	Not specified	Female nursing students, mean age 21.1 ± 1.2; 18–24 years (98.2% in 21–24 age group)	Premenstrual Symptoms Screening Tool (PSST)	114	28.1% (moderate–severe PMS)	6.10%	Not reported
Jose et al. (14)	Kerala, India	Mixed-method: descriptive cross-sectional+ focus group discussion (FGD)	2021	Female nursing students, 19–21 (all 21)	Stainer and Wilkin PMS criteria; FGD guide	100 (quant), 10 (qual)	86% (24% mild, 54% moderate, 8% severe)	Not assessed	Not reported
Wuni et al. (16)	Northern Ghana	Descriptive cross-sectional, structured questionnaire	Sept–Oct 2022	Female nursing and midwife trainees, mean age 22.6 ± 2.4 (range 18–49; majority 20–24)	Structured/validated tool, own instrument	303	75.6% (PMS, occasional+yes)	Not reported	66.70%
Mahmood KI (26)	Erbil, Iraq	Quantitative cross-sectional, online self-report	Nov 2022—Jan 2023	Female nursing and midwifery students, mean age 20.8 ± 1.9; BMI 22.4	ACOG PMS criteria, custom knowledge/attitude/impact scale	222	53.60%	Not reported	62.60%
Nagalakshmi and Tamizharasi (27)	Salem, India	Descriptive; semi-structured interviews/checklist	Not specified	18–22	PMS checklist; rating scale on menstrual practices	156	85.3% (133/156) had PMS; 14.7% (23/156) did not	Not reported	68 (44%)
Singh et al. (2022) (15)	Jodhpur, India	Descriptive cross-sectional; semi-structured questionnaire	Not stated	Nursing students, mean age 21.04 ± 1.4, mean menarche 13.69 ± 1.38	Custom; includes lifestyle, PMS, dysmenorrhea	98	70.40%	Not reported	77.60%
Çoban et al. (28)	Antalya, Turkey	Cross-sectional, validated surveys	Feb–Apr 2020	Female nursing students, mean age ∼20.5 (SD ≈1.4)	Premenstrual Symptoms Screening Tool (PSST), EAT-26, TFEQ-R18	504	44% (moderate–severe PMS); 40% mild/no PMS	15.9% (PMDD)	Not directly stated, but 60%+ reported using analgesics for pain
Kim and Park (29)	Seoul, S. Korea	Two-stage: (1) retrospective cross-sectional; (2) prospective cohort	Apr–Jun 2017 (Stage 1); May–Sep 2017 (Stage 2)	Female nursing students, mean age 20.6 (range 18–25)	Daily Record of Severity of Problems (DRSP); PBAC	151 (stage 1); 17 (stage 2)	42.4% (retrospective DRSP); 22.4% (clinically confirmed by prospective DRSP)	Not reported	37.4% menorrhagia (PBAC ≥100)
Kustriyanti and Rahayu (30)	Semarang, Indonesia	Cross-sectional, self-report, WHOQOL-BREF	July–August 2017	Nursing students, mean age 19.17 ± 1.1 (17–26); menarche mean 12.98 ± 1.5	ACOG PMS, DSM-IV PMDD, WHOQOL-BREF	207	60.8% (126/207)	31.4% (65/207)	54%
Abreu-Sánchez et al. (31)	Huelva, Spain	Cross-sectional, paper self-report questionnaire	Dec 2019–Jan 2020	Female nursing students, mean age 21.1 ± 2.4; menarche 12.2 ± 1.5	Ad hoc questionnaire, VAS (pain), symptom checklist	354	Not reported	Not reported	73.8% (total); 63.3% primary, 10.5% secondary dysmenorrhea
Vlachou et al. (32)	Athens, Greece	Cross-sectional, self-report questionnaire	May–Oct 2017	Nursing students, mean age 23.9 (range 18–55), menarche mean 12.7	Custom questionnaire; VAS (pain)	631	Not reported	Not reported	89.2% (*n* = 563); severe pain 52.5%, moderate 33.9%, mild 13.6%
Fernández-Martínez et al. (33)	Ciudad Real, Spain	Cross-sectional, anonymous self-report	May–June 2017	Female nursing students, mean age 20.6 (SD 3.3), menarche mean 12.5 (SD 1.5)	Custom questionnaire, VAS pain	258	Not reported	Not reported	74.8% (*n* = 193, 95% CI: 69.5–80.1%)
Akhtar et al. (34)	Peshawar, Pakistan	Cross-sectional, observer-rated PTMS scale	Mar–Jun 2017	Nursing students, mean age 22.4 ± 3.8; menarche 13.9 ± 1.4	Revised Premenstrual Tension Syndrome Scale (PTMS-OR)	200	51% (*n* = 102); 41% mild, 47% moderate, 12% severe	Not reported	Not reported
Abirami and Ambika (35)	Chennai, India	Descriptive, self-administered questionnaire	Jan 5–21, 2015	Adolescent girls, 17–26 years (51% age 17–19, 46% age 20–24)	Custom PMS questionnaire, by study author	100	100% (all had PMS: 26% mild, 55% moderate, 19% severe)	Not reported	Excluded
Cetin et al. (36)	Turkey	Cross-sectional, face-to-face interviews	Nov 2015–Mar 2016	Nursing students, mean age 21.4 (18–29)	Premenstrual Syndrome Scale (PMSS, validated)	102	70% (PMSS ≥111)	Not reported	Not reported
Osman and El-Houfey (37)	Egypt	Cross-sectional, self-report	Oct–Nov 2014	Nursing students, mean age 20 (17–22)	Self-developed questionnaire, Quality of Life Scale, Verbal Multidimensional Scoring System (validated, *α*=0.95)	188	Not reported	Not reported	90.40%
Shewte and Sirpurkar (6)	India	Cross-sectional, self-report	Nov 2014–Jan 2015	Nursing students, mean 19.2 years	SF-36 (validated, HRQoL), custom questionnaire	109	Not reported	Not reported	69 (63.3%)

Several of the included studies provided additional details on the academic and social implications of menstrual disturbances, along with the interventions attempted and the authors' concluding remarks. A summary of these findings is presented in Table 2.

**Table 2:** Summary of included studies reporting academic and social impacts, interventions, and conclusions.

Author (Year)	Impact on academic and social life	Intervention to reduce menstrual disturbance	Article conclusion
Akalin and Karpuzluk (2025) (12)	Not directly quantified, but PMS associated with negative effects on daily functioning, performance, social relationships, and QOL; higher depression, anxiety, and stress scores	None tested in study; recommendations for holistic and tailored approaches considering lifestyle, mental health, menstrual health	PMS is common among nursing students, highly related to mental health, lifestyle, and psychological factors; interventions should address multiple dimensions
Tekbaş and Güder (2024) (13)	Not directly measured, but authors state PMS “reduces quality of life and academic achievement”; high rates of moderate-to-severe PMS; PMS more severe in those with irregular cycles/dysmenorrhea	48.1% used non-pharmacological methods (rest, hot packs, sleep, herbal remedies, music, yoga, aromatherapy); 33.2% used medications; low rates sought professional help	Most students had moderate/severe PMS and coped mainly via self-care, but few sought medical advice. Education about professional support and healthy coping needed
Qutishat et al. (2024) (24)	High PMS prevalence; 14.6% “always” and 35.9% “sometimes” reported impaired work performance; common symptoms included anxiety, irritability, social withdrawal, and concentration issues; negative effect on QOL and academic functioning discussed	None tested in study; observed a positive attitude toward seeking professional psychological help among students with more severe PMS	PMS is highly prevalent and associated with academic and psychosocial impairment. Attitudes toward seeking psychological help are positive, especially with higher PMS severity; mental health services should be accessible
Lone and Singh (2024) (25)	81.3% of those with moderate–severe PMS and 100% with PMDD had impaired college productivity/efficiency; emotional wellbeing and behavior significantly affected	None tested in study; educational session was conducted after survey for awareness but not evaluated as intervention	PMS and PMDD are common and cause substantial academic, behavioral, and emotional impairment. Timely recognition and management in college health programs is essential
Jose et al. (2024) (14)	PMS identified as a major cause of absenteeism and reduced academic performance; frequent mentions of academic overload, exam stress, and fear of not completing requirements; focus group revealed both psychological and lifestyle burdens	None tested in study; educational motivation for PMS management is recommended	PMS is highly prevalent and shaped by intrinsic (biological, emotional) and extrinsic (lifestyle, academic, sociocultural) factors. Ongoing education and motivation are needed to reduce its academic and psychological impact
Wuni et al. (2023) (16)	62.4% reported negative impact on academics; 77.2% poor concentration in class; 35.6% missed classes due to dysmenorrhea; 54% reported social life affected; many unable to perform normal activities	Most (47.5%) did nothing; 20.8% used medications (mainly antispasmodics/NSAIDs); 13.4% used non-pharmacological methods (sleep, hot compress); 18.3% used both	High prevalence and severity of dysmenorrhea among nursing and midwifery trainees, with major academic and social disruption; self-management is common, but professional help is rarely sought
Mahmood et al. (2023) (26)	70.3% reported PMS disturbs daily routine; 36.9% missed college due to PMS; 67.1% lacked motivation, 62.2% lacked concentration, 45% reported low academic scores	Self-management only: most commonly sleep, hot drinks, hot pack; no formal interventions or professional help	PMS is common and disrupts daily and academic life; knowledge was generally good, but perception of PMS often did not match diagnostic criteria
Nagalakshmi and Tamizharasi (2023) (27)	51% had impaired work activities during menstruation; irritability, headache, anxiety, tension, depression, and anger were common symptoms affecting daily functioning	None tested; study observed menstrual hygiene and practice only; all had “adequate” practice by study's scale	PMS is highly prevalent and impairs work and daily activities among adolescent girls; no demographic or clinical variable was associated with PMS level
Singh et al. (2022) (15)	12.2% reported college absenteeism due to menstrual problems; 51% reported work performance moderately or severely affected; dysmenorrhea main cause for absenteeism	None tested; study was observational only; self-management not detailed	Despite medical background, many nursing students experience PMS and dysmenorrhea that negatively affect academic performance; lifestyle factors not significantly associated
Çoban et al. (2021) (28)	Both PMS and especially PMDD groups reported increased impairment in daily, social, and academic function; disordered eating behaviors more common in PMDD	None tested; self-management and coping methods not detailed	PMDD is more severe than PMS and is strongly associated with disordered eating, psychiatric comorbidity, and higher impairment in function
Kim and Park (2020) (29)	PMS linked to decreased quality of life and academic/social achievement; impact discussed but not directly quantified in terms of absenteeism or performance	None tested; study was observational; no interventions evaluated	PMS prevalence varies depending on method of measurement; prospective tracking gives lower estimates than retrospective reporting; PMS affects QOL
Kustriyanti and Rahayu (2020) (30)	70% reported PMS disturbs learning; >50% poor concentration, absenteeism, failed exams; significant reduction in all domains of quality of life (QOL)	49.8% did nothing; 18.4% took medication; 15.5% used herbs; 4.3% used massage; self-management only, no formal intervention studied	PMS and PMDD are common and significantly reduce academic achievement and QOL among nursing students; most students do not seek medical help
Abreu-Sánchez et al. (31)	Absenteeism and reduced academic/social performance reported, especially in those with severe pain; impact not quantified but implied as significant	None formally tested; observational only; self-management patterns not detailed	Dysmenorrhea is common among nursing students; secondary dysmenorrhea associated with greater menstrual symptoms and should prompt further investigation
Vlachou et al. (2019) (32)	Severe dysmenorrhea led to increased absenteeism from classes and reduced participation in studies, exercise, and social activities; pain severity closely linked to impairment	71.4% used pain relievers (NSAIDs, paracetamol); non-pharmacologic interventions not detailed	Dysmenorrhea is highly prevalent and can severely impact students’ wellbeing and participation; early menarche and family history increase risk
Fernández-Martínez et al. (2018) (33)	75.6% with dysmenorrhea reported impaired daily activities; moderate/severe pain frequent; absenteeism not directly quantified	92% used self-medication (analgesics); non-pharmacological interventions not described	Primary dysmenorrhea is highly prevalent and significantly impairs daily function; risk factors include diet, family history, and cycle duration
Akhtar et al. (2017) (34)	PMS impaired interest in daily/academic activities (81%), irritability (78%), mood changes, concentration, anxiety, depression	None tested; observational only; coping methods not described	PMS is common among nursing students, mainly causing psychological symptoms and functional impairment; most cases were mild or moderate in severity
Abirami and Ambika (2017) (35)	PMS described as “disturbing daily life”; impact not quantified, but all participants reported experiencing PMS symptoms	None assessed during study; all participants received self-instructional PMS information after survey (not evaluated)	PMS is highly prevalent among adolescent nursing students and causes substantial disturbance; no demographic or clinical variables linked to PMS severity
Cetin et al. (2022) (36)	PMS negatively affected academic life, productivity, social relations, and quality of life. Higher PMSS scores linked to unhealthy diet, negative mood, and less use of coping	Most effective: dietary changes, increased fluid intake, warm showers, psychological support. Also assessed: relaxation, exercise, herbal, vitamins, massage, OCs, medication, yoga (less effective)	Among nursing students, dietary changes, increased fluid intake, psychological support, and warm showers most effectively reduced PMS severity. Interventions to improve knowledge and coping strategies are recommended to enhance wellbeing and reduce PMS impact
Osman and El-Houfey (2016) (37)	Severe dysmenorrhea associated with 4× risk of college absence, reduced academic performance, restricted social and physical activities	Rest, OTC meds, herbal drinks, hot tea, heating pad, exercise (low uptake)	Dysmenorrhea is highly prevalent; has strong negative effects on QOL, daily life, and academics. Physical activity and health education are recommended to reduce burden
Shewte and Sirpurkar (2016) (6)	Dysmenorrhea led to lower HRQoL in all domains; more absenteeism, poor concentration, restricted activities in nursing students	Allopathic drugs, hot fomentation, most did not use any method	Dysmenorrhea highly prevalent (63.3%) among nursing students; severity impacts HRQoL; interventions and awareness are needed

Quality appraisal using the JBI Critical Appraisal Checklist for Prevalence Studies showed that most studies scored moderate to high in methodological quality. Common limitations included reliance on self-reported symptoms and variation in diagnostic criteria for PMS, PMDD, and dysmenorrhea (Supplementary Appendix A, Supplementary JBI data) (Table 3).

**Table 3:** Joanna Briggs Institute (JBI) critical appraisal results for included studies.

Author (Year)	Q1	Q2	Q3	Q4	Q5	Q6	Q7	Q8	Q9	Overall risk of bias
Akalin and Karpuzluk (2025) (12)	Yes	Yes	Yes	Yes	Yes	Yes	Yes	Yes	Yes	Low
Tekbaş and Güder (2024) (13)	Yes	Yes	Yes	Yes	Yes	Yes	Yes	Yes	Yes	Low
Qutishat et al. (2024) (24)	Yes	Yes	Yes	Yes	Yes	Yes	Yes	Yes	Yes	Low
Lone and Singh (2024) (25)	Yes	Yes	Yes	Yes	Yes	Yes	Yes	Yes	Yes	Low
Jose et al. (2024) (14)	Yes	Yes	Yes	Yes	Yes	Yes	Yes	Yes	Yes	Low
Wuni et al. (2023) (16)	Yes	Yes	Yes	Yes	Yes	Yes	Yes	Yes	Yes	Low
Mahmood et al. (2023) (26)	Yes	Yes	Yes	Yes	Yes	Yes	Yes	Yes	Yes	Low–moderate
Nagalakshmi and Tamizharasi (2023) (27)	Yes	Yes	Yes	Yes	Yes	No	Yes	Yes	Yes	Moderate
Singh et al. (2022) (15)	Yes	Yes	Yes	Yes	Yes	No	Yes	Yes	Yes	Moderate
Çoban et al. (2021) (28)	Yes	Yes	Yes	Yes	Yes	Yes	Yes	Yes	Yes	Low
Kim and Park (2020) (29)	Yes	Yes	Yes	Yes	Yes	Yes	Yes	Yes	Yes	Low
Kustriyanti and Rahayu (2020) (30)	Yes	Yes	Yes	Yes	Yes	Yes	Yes	Yes	Yes	Low
Abreu-Sánchez et al. (31)	Yes	Yes	Yes	Yes	Yes	Yes	Yes	Yes	Yes	Low–moderate
Vlachou et al. (2019) (32)	Yes	Yes	Yes	Yes	Yes	Yes	Yes	Yes	Yes	Low–moderate
Fernández-Martínez et al. (2018) (33)	Yes	Yes	Yes	Yes	Yes	Yes	Yes	Yes	Yes	Low–moderate
Akhtar et al. (2017) (34)	Yes	Yes	Yes	Yes	Yes	Yes	Yes	Yes	Yes	Low
Abirami and Ambika (2017) (35)	Yes	Yes	Yes	Yes	Yes	No	Yes	Yes	Yes	Moderate
Cetin et al. (2022) (36)	Yes	Yes	Yes	Yes	Yes	Yes	Yes	Yes	Yes	Low
Osman and El-Houfey (2016) (37)	Yes	Yes	Yes	Yes	Yes	Yes	Yes	Yes	Yes	Low–moderate
Shewte and Sirpurkar (2016) (6)	Yes	Yes	Yes	Yes	Yes	Yes	Yes	Yes	Yes	Low–moderate

### 3.3 Thematic synthesis

The qualitative synthesis of the included studies identified six overarching themes (Table 4). First, the high prevalence of menstrual disorders was consistently associated with adverse academic and social impacts, including absenteeism, reduced concentration, and difficulties in clinical training. Second, students relied heavily on self-management strategies such as over-the-counter analgesics, rest, or herbal remedies, while formal help-seeking remained rare due to stigma and normalization of symptoms. Third, the disorders imposed a significant psychological and quality of life burden, with many students reporting heightened stress, anxiety, irritability, and diminished daily functioning. Fourth, multiple risk factors and predictors were identified, including lifestyle patterns (diet, sleep, and stress), body mass index, and family history of menstrual problems. Fifth, the review highlighted that institutional or structured interventions were largely absent, leaving students to adopt mostly self-directed or peer-shared strategies. Finally, several methodological notes emerged, including heterogeneity in diagnostic tools, reliance on self-reported measures, and inconsistent definitions of PMS, PMDD, and dysmenorrhea, underscoring the need for standardized approaches in future research.

**Table 4:** Thematic synthesis of included studies.

Theme	Key Findings/Examples	Articles
1. High Prevalence and Academic/Social Impact	Almost every study highlights a *high prevalence* of PMS, dysmenorrhea, or PMDD among nursing students (often >50%) Academic impact includes absenteeism, poor concentration, reduced motivation, failed exams, and impaired work performance Social impact includes withdrawal, irritability, and reduced participation in daily activities	Akalin and Karpuzluk (2025) (12); Tekbaş and Güder (2024) (13); Qutishat et al. (2024) (24); Lone and Singh (2024) (25); Jose et al. (2024) (14); Wuni et al. (2023) (16); Mahmood et al. (2023) (26); Nagalakshmi and Tamizharasi (2023) (27); Singh et al. (2022) (15); Çoban et al. (2021) (28); Kim and Park (2020) (29); Kustriyanti and Rahayu (2020) ) (30); Abreu-Sánchez et al. (36) (31); Vlachou et al. (2019) (32); Fernández-Martínez et al. (2018) (33); Akhtar et al. (2017) (34); Abirami and Ambika (2017) (35)
2. Self-Management and Low Formal Help-Seeking	Students primarily use self-management for menstrual symptoms (rest, hot packs, sleep, herbal remedies, over-the-counter analgesics) Very low rates of consulting healthcare professionals or using formal interventions even when symptoms are severe Some studies found a positive attitude toward psychological help, but little actual service use	Tekbaş and Güder (2024) (13); Wuni et al. (2023) (16); Mahmood et al. (2023) (26); Kustriyanti and Rahayu (2020) (30); Fernández-Martínez et al. (2018) (33); Kim and Park (2020) (29); Abirami and Ambika (2017) (35); Nagalakshmi and Tamizharasi (2023) (27); Qutishat et al. (2024) (24)
3. Psychological and Quality-of-Life (QOL) Burden	Menstrual disorders are closely associated with psychological symptoms (depression, anxiety, stress, low motivation, emotional lability) Students with PMS/PMDD have significantly lower QOL scores in physical, psychological, social, and environmental domains Comorbidity with disordered eating (especially PMDD) and psychiatric symptoms is highlighted in some articles	Akalin and Karpuzluk (2025) (12); Çoban et al. (2021) (28); Mahmood et al. (2023) (26); Lone and Singh (2024) (25); Kustriyanti and Rahayu (2020) (30); Vlachou et al. (2019) (32)
4. Risk Factors and Predictors	Several studies discuss risk factors: family history, early menarche, diet (e.g., cola, meat), physical inactivity, BMI, sleep quality, and stress Lifestyle factors like poor diet, lack of exercise, and high stress are consistently associated with more severe symptoms Some studies found no direct link between demographics and PMS severity, highlighting complexity	Jose et al. (2024) (14); Fernández-Martínez et al. (2018) (33); Abreu-Sánchez et al. (36) (31); Akalin and Karpuzluk (2025) (12); Singh et al. (2022) (15); Kustriyanti and Rahayu (2020) (30); Vlachou et al. (2019) (32)
5. Interventions; Mainly Absent or Self-Directed	Only a minority of studies discuss any structured intervention (e.g., psychoeducation post-survey, educational motivation, or recommendations for future programs) Yoga, mindfulness, pharmacological trials, supplements, and other interventions are largely absent from these studies Most recommend future education or college-based programs	Jose et al. (2024) (14); Akalin and Karpuzluk (2025) (12); Tekbaş and Güder (2024) (13); Lone and Singh (2024) (25); Abirami and Ambika (2017) (35) (education after survey); all others recommend but do not test interventions
6. Methodological Notes and Tools	Many studies use validated screening/diagnostic tools (ACOG, PMSS, PSST, DRSP, VAS, etc.) Some discuss the difference between retrospective and prospective diagnosis of PMS/PMDD [e.g., Kim and Park (2020) (29)]	See Table 1

In addition to the thematic synthesis, a comparative table was developed to map how individual studies addressed the academic and social impact of menstrual disturbances, the interventions (if any) employed, and the conclusions drawn. Across studies, menstrual disorders were consistently linked to academic disruption, including reduced classroom engagement, absenteeism during examinations, and impaired participation in clinical placements. Social functioning was similarly affected, with students reporting withdrawal from daily activities and interpersonal interactions. The reported interventions were limited and varied, ranging from self-administered pharmacological treatments (such as analgesics and oral contraceptives) to non-pharmacological approaches (including exercise, dietary changes, herbal remedies, and rest). Notably, very few studies described formal institutional measures such as wellness clinics, screening programs, or structured support services. Most conclusions emphasized the high prevalence and under-addressed burden of menstrual disorders and called for greater awareness, institutional support, and evidence-based interventions to mitigate their academic and psychosocial impact.

### 3.4 Prevalence estimates (meta-analysis)

#### 3.4.1 Dysmenorrhea

**Figure 2:**
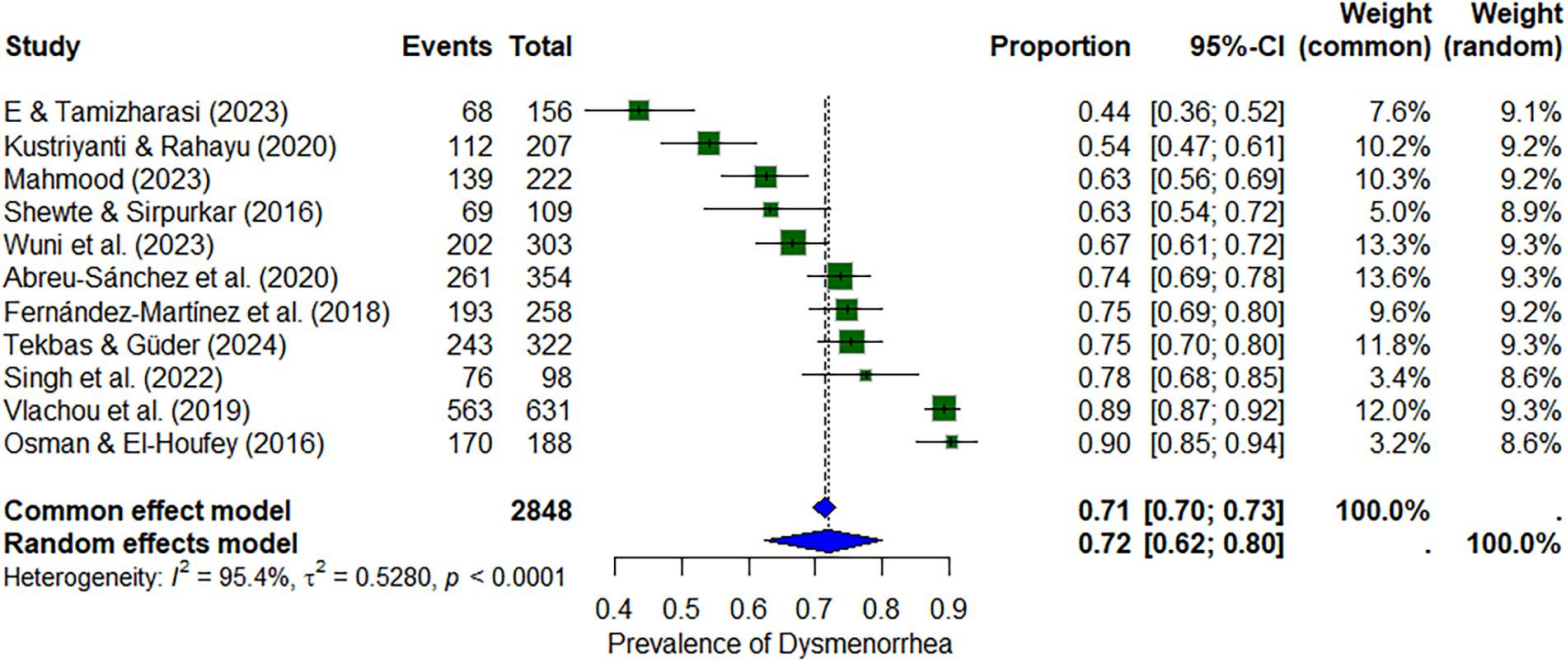
Forest plot of dysmenorrhea prevalence among nursing students.

Across 10 studies (*n* ≈ 2,848), the pooled prevalence of dysmenorrhea was 72% (95% CI: 62%–80%) under the random-effects model, with significant heterogeneity (*I*^2^ = 95.4%). Prevalence estimates ranged from 44% to 90% (27, 37) (Figure 2).

#### 3.4.2 Premenstrual syndrome

**Figure 3:**
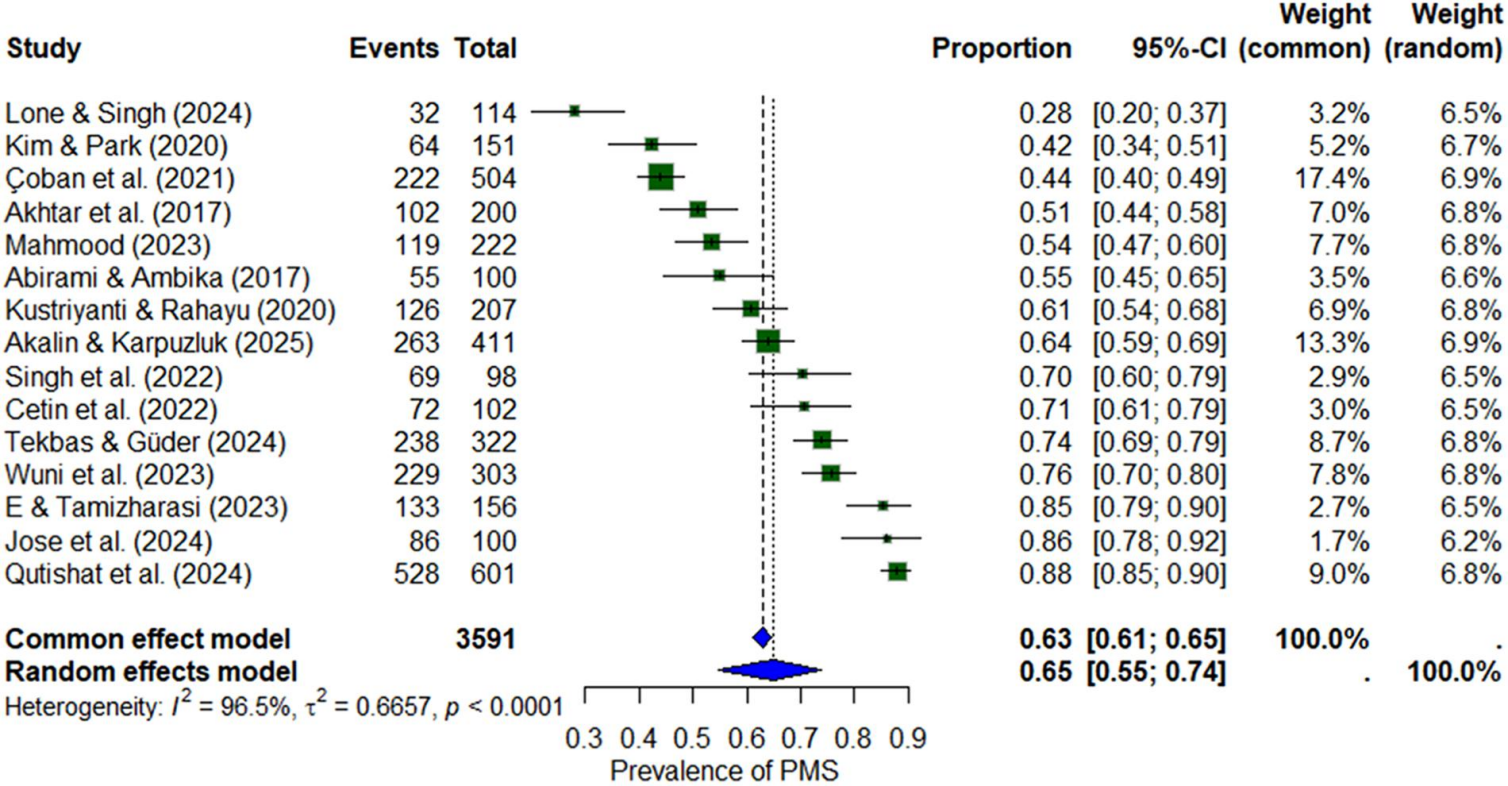
Forest plot of premenstrual syndrome (PMS) prevalence among nursing students.

Fourteen studies (*n* ≈ 3,591) reported the prevalence of PMS. The pooled prevalence under the random-effects model was 65% (95% CI: 55%–74%), with substantial heterogeneity (*I*^2^ = 96.5%). Reported prevalence ranged widely, from as low as 28% to as high as 88% (24, 25) (Figure 3).

#### 3.4.3 Premenstrual dysphoric disorder

**Figure 4:**
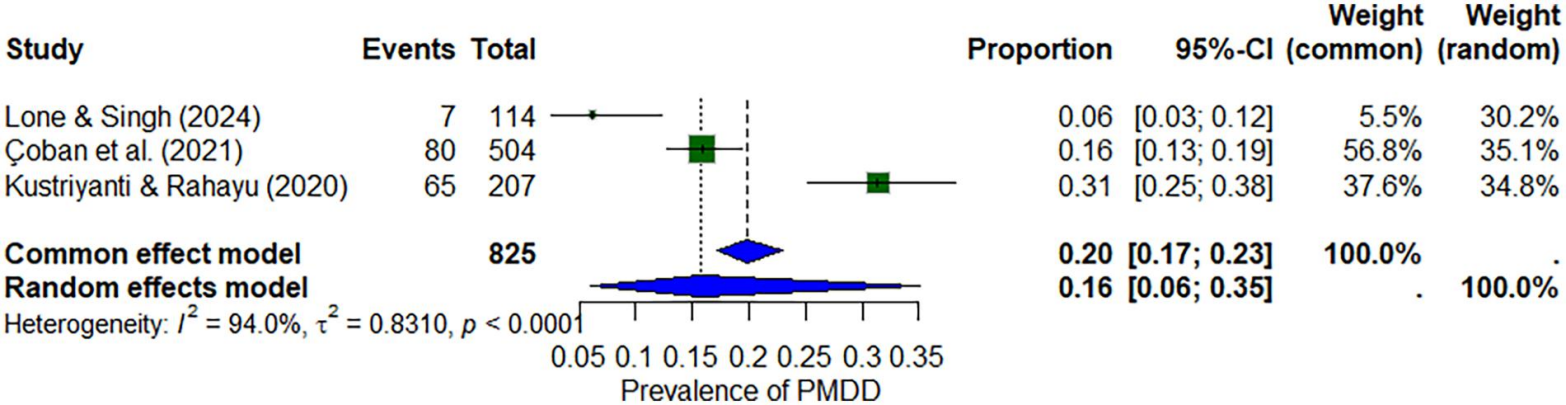
Forest plot of premenstrual dysphoric disorder (PMDD) prevalence among nursing students.

Three studies (*n* ≈ 825) investigated PMDD. The pooled prevalence was 16% (95% CI: 6%–35%), with heterogeneity of *I*^2^ = 94%. Individual study estimates ranged from 6% to 31% (Figure 4).

#### 3.4.4 Combined PMDD/severe PMS

**Figure 5:**
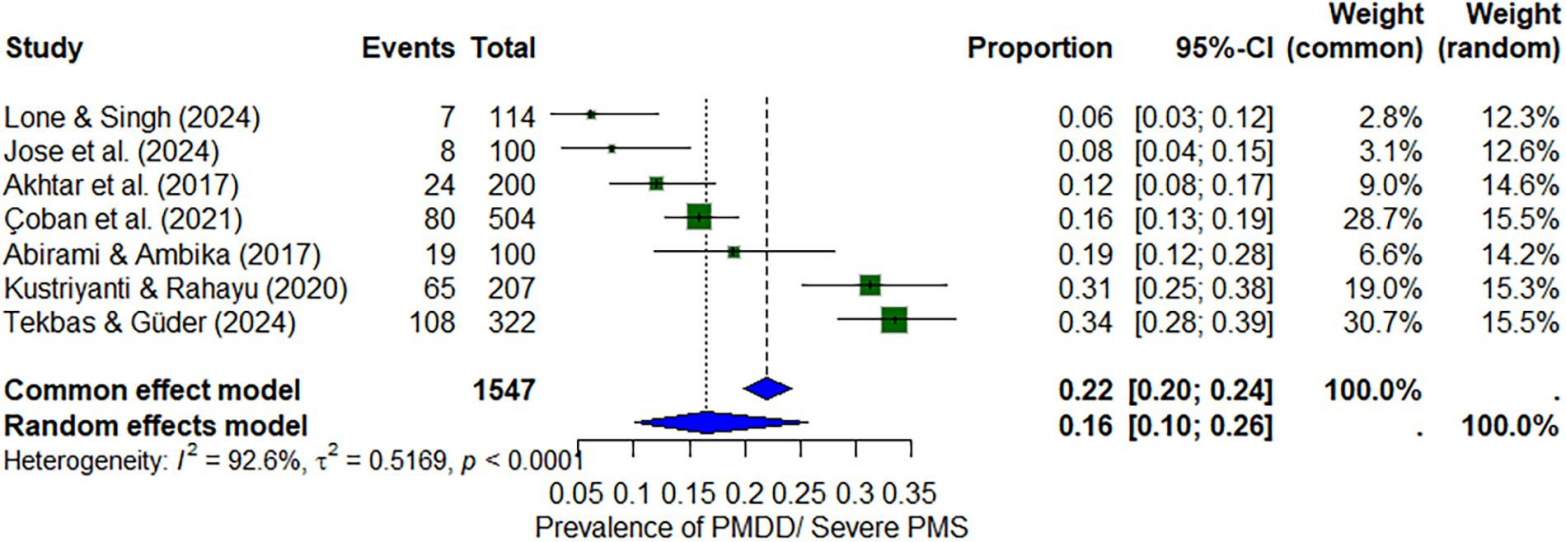
Forest plot of combined severe PMS and PMDD prevalence among nursing students.

Six studies (*n* ≈ 1,547) reported the prevalence of either PMDD or severe PMS, combined for comparability. The pooled prevalence was 16% (95% CI: 10%–26%), with heterogeneity of *I*^2^ = 92.6% (Figure 5).

### 3.5 Publication bias

Funnel plots for each outcome (dysmenorrhea, PMS, PMDD, and Combined PMDD/Severe PMS) are provided in Supplementary Figures 1–4. Visual inspection suggested a reasonably symmetrical distribution of studies around the pooled effect size, although some asymmetry was noted in smaller studies, particularly for PMS. This indicates a potential small-study effect, but there is no firm evidence of systematic publication bias. Formal Egger's regression tests were not conducted due to the limited number of included studies per outcome (<10 in several cases), consistent with recommendations to interpret funnel plots cautiously under such conditions.

## 4 Discussion

The present systematic review and meta-analysis provide the most up-to-date synthesis of the prevalence, academic and social implications, and coping strategies for PMS, PMDD and dysmenorrhea among nursing students.

PMS prevalence among university women varies widely by setting, but consistently remains high. In Ethiopia, PMS was reported by 37.9% of students at Wolkite University (38). Among Indian cohorts, estimates were higher: In nursing-only samples, PMS prevalence reached approximately 78% (15) and 74% in another nursing college sample (35). Across India more broadly, a national meta-analysis reported pooled PMS prevalence of 43% (39).

A smaller but clinically important subset met criteria for PMDD: 6.1% among Indian nursing students (25), 15.9% in Turkish nursing students using the Premenstrual Symptoms Screening Tool (PSST) (28), and 31% in Indonesian health science students (30).

Dysmenorrhea was the most common disturbance. In Greek nursing students, prevalence was 89% (32); similar studies in Spain and Ghana reported 75% and 67%, respectively (16, 33).

### 4.1 Academic and social impact/help-seeking

Across settings, menstrual disorders impaired learning and daily functioning. In Spain, nursing students with dysmenorrhea had nearly sevenfold higher odds of absenteeism (OR 6.95, 95% CI 3.39–14.25), with dizziness, nausea/vomiting, and sleep problems further increasing risk (40). A companion study documented high presenteeism (92.7%) and substantial interference with study and clinical tasks (31). Consistent patterns appeared elsewhere: Ghanaian nurse/midwife trainees reported impaired concentration during lectures (77%) and limitations in normal physical activity (58%), with many resorting to antispasmodics (16). Among Indian nursing students, PMS was linked to reduced motivation and attentional difficulties (10, 15).

Help-seeking was typically low. Spanish nursing students described normalization of menstrual pain, leading to self-medication with NSAIDs and reluctance to consult clinicians (17). In Ethiopia, 93% reported coping on their own, most commonly rest and sleep, with family history, severe pain, early menarche, long menses, and irregular cycles predicting PMS (38). Similar patterns were observed in India and Indonesia, where students frequently used hot packs, warm drinks, and OTC analgesics, but rarely accessed professional care (30, 35).

### 4.2 Definition, classification, and rationale for combining severe PMS with PMDD

The distinction between PMS and PMDD has long been debated, reflecting both diagnostic uncertainty and methodological inconsistency (4). PMS is commonly defined as the cyclical occurrence of affective, behavioral, and physical symptoms during the luteal phase, severe enough to interfere with daily functioning (ACOG, 2015). PMDD, as codified in the DSM-5, represents the most severe end of the spectrum, requiring at least five symptoms, one of which must be affective (e.g., mood swings, irritability, depressed mood), with clear impairment of social or occupational functioning (41).

In practice, however, few prevalence studies among nursing students apply full DSM-5 criteria prospectively. For example, researchers identified PMDD in 15.9% of Turkish nursing students using the PSST, while also reporting that nearly half experienced “moderate-to-severe” PMS (28). Similarly, another study found 31% PMDD prevalence in Indonesian students, but acknowledged that many of these cases overlapped with their “severe PMS” group (30). In contrast, other studies reported high rates of moderate-to-severe PMS (55% and 19%) without applying a PMDD classification, effectively capturing similar functional impairment under a different label (35).

This overlap has been widely recognized in the broader literature, with researchers noting that many students with “severe PMS” experience symptom severity and functional impairment equivalent to PMDD, but are classified differently due to tool variability or recall bias (42). Functional impairment, rather than absolute symptom count, may be the most clinically meaningful marker in young populations such as nursing students (43).

Given these diagnostic challenges and the consistent academic and psychosocial disruption documented in our review, we grouped “severe PMS” and PMDD into a single analytical category for pooled prevalence estimates. This approach aligns with recommendations from recent meta-analyses and ensures that the true burden of clinically significant premenstrual distress is not underestimated by rigid diagnostic separation (44). While PMS and PMDD remain distinct in formal classifications, our combined analytic approach better reflects the lived experience of nursing students, for whom functional consequences are paramount.

### 4.3 Psychological and quality-of-life (QOL) burden

Menstrual disorders among nursing students impose a substantial burden on psychological wellbeing, quality of life, and academic functioning. Across the 20 included studies, prevalence rates of PMS, PMDD, and dysmenorrhea were consistently high, with symptoms often severe enough to interfere with concentration, clinical performance, and social participation (12, 16, 35). Students frequently reported depressive mood, irritability, fatigue, and anxiety, all of which are recognized risk factors for poorer mental health trajectories and diminished academic resilience (10, 25). The strong association between menstrual disorders and psychological distress has also been highlighted in global epidemiological analyses, which estimate that PMS affects nearly half of women of childbearing age worldwide and contributes substantially to years lived with disability (7, 8).

The quality-of-life impact extends beyond mental health to academic and social domains. Several included studies documented high rates of absenteeism from lectures, reduced efficiency during clinical placements, and impaired academic scores among those with severe PMS or PMDD (13–15). Dysmenorrhea, often described as incapacitating, further limited students' participation in sports, exercise, and social activities, leading to isolation and strained peer relationships (32, 33). These findings align with earlier systematic reviews in medical students, which reported PMS prevalence above 50% and dysmenorrhea prevalence exceeding 70%, both strongly correlated with stress, absenteeism, and academic underperformance (18).

Taken together, the evidence underscores that menstrual disorders are not transient inconveniences but significant health challenges with profound psychosocial and academic consequences. For nursing students, who already face intense educational and clinical demands, these conditions can amplify stress, reduce coping capacity, and compromise professional development.

### 4.4 Explanatory factors and mechanisms

The consistently high prevalence of PMS, PMDD, and dysmenorrhea among nursing students reflects a complex interplay of biological, psychological, and social factors. At the biological level, cyclical hormonal fluctuations, particularly changes in estrogen and progesterone, modulate serotonergic and GABAergic pathways, influencing mood, cognition, and pain sensitivity (4, 45). The neurosteroid allopregnanolone, a metabolite of progesterone, has been implicated in PMDD due to its paradoxical anxiogenic effect in sensitive individuals (46). Dysmenorrhea, by contrast, is largely driven by endometrial overproduction of prostaglandins, leading to heightened uterine contractility and ischemia, explaining the incapacitating physical pain often reported in this group (31, 33). Genetic predisposition may also play a role, with several studies noting higher prevalence in students with a positive family history of menstrual disorders (15, 32).

Beyond biology, nursing students are uniquely vulnerable due to educational and lifestyle factors. Heavy academic workloads, long clinical shifts, and irregular sleep schedules exacerbate both physical and psychological symptoms. Some researchers have reported that over 70% of students experiencing PMS described disrupted sleep quality, while others found that dysmenorrhea significantly impaired classroom concentration and clinical performance (10, 16). Lifestyle behaviors such as smoking, alcohol, and high caffeine consumption have been associated with higher PMS prevalence, while protective behaviors like regular physical activity and balanced diets remain underutilized (12, 15). These findings echo broader epidemiological research linking obesity, sedentary lifestyle, and poor dietary quality to more severe menstrual disturbances (47–50).

Sociocultural and psychosocial mechanisms further contribute to the burden. Several included studies highlighted the role of stigma and normalization in delaying help-seeking, with many students accepting PMS or dysmenorrhea as “part of womanhood” rather than as treatable health concerns (24, 35). This aligns with evidence from global reviews confirming that menstrual stigma discourages disclosure and fosters reliance on self-care or informal remedies instead of professional consultation (5). Psychological distress, including anxiety and depression, may both result from and exacerbate menstrual symptoms, creating a vicious cycle that is particularly problematic in student populations already at high risk for burnout (25, 51).

The “Biopsychosocial Model” offers a useful framework for interpreting these findings. Biological drivers such as prostaglandin overproduction or neurohormonal sensitivity interact with psychological vulnerabilities like stress, depression, and sleep disturbance, while social influences such as stigma, lack of institutional support, and cultural norms shape symptom perception and reporting (19, 45). For example, in Spain, researchers found that dysmenorrhea severity correlated not only with menstrual physiology but also with reported stress and coping strategies of students, underscoring the interdependence of these domains (31, 52).

Similarly, the “Health Belief Model” helps explain patterns of self-management and low formal help-seeking observed in our review (20). According to this model, students' decisions to seek care are shaped by perceived severity of symptoms, perceived benefits of intervention, perceived barriers (such as stigma, cost, or lack of access), and cues to action. Studies from Turkey and Oman illustrate this: While prevalence was high, few students sought medical advice, citing normalization and lack of awareness of effective treatments (13, 24). The reliance on non-pharmacological self-care measures such as rest, hot drinks, or applying warm packs reflects not only resource constraints but also cultural perceptions of menstruation as a private issue not warranting clinical intervention (10, 16).

Taken together, these explanatory mechanisms highlight why nursing students may represent a uniquely high-risk group compared to other university populations. Their biological vulnerability is compounded by demanding academic environments, insufficient institutional support, and sociocultural attitudes that normalize suffering and discourage proactive health-seeking. Applying theoretical frameworks such as the Biopsychosocial and Health Belief Models underscores the multifactorial nature of menstrual disorders and highlights the importance of multi-level solutions—biological, psychological, and social—to adequately address the burden.

### 4.5 Interventions and management approaches

Our review revealed that while menstrual disorders are pervasive among nursing students, interventions remain fragmented, largely self-directed, and inadequately integrated into institutional or health system frameworks. Across the 20 included studies, students commonly reported resorting to rest, hot drinks, warm packs, sleep, and reduced activity as first-line coping strategies, reflecting the normalization of symptoms and limited access to formalized care (10, 13, 16). Pharmacological approaches such as analgesics (NSAIDs, antispasmodics) were used by some participants, but reliance on informal, non-prescription methods far outweighed engagement with structured clinical management (32, 33). Few studies documented proactive medical consultation or targeted institutional support for menstrual health.

Evidence from the broader literature demonstrates that a wide spectrum of interventions can effectively reduce the burden of PMS, PMDD, and dysmenorrhea. Lifestyle interventions such as exercise, yoga, and mindfulness-based stress reduction have shown significant benefits in randomized controlled trials (RCTs), improving both symptom severity and psychological outcomes (18, 53, 54). Dietary modification—particularly adherence to a Mediterranean diet or supplementation with omega-3 fatty acids—has also been associated with reduced prevalence and intensity of symptoms (47).

Pharmacological therapies remain central in evidence-based management. NSAIDs are recommended first-line treatment for dysmenorrhea due to their ability to reduce prostaglandin synthesis and relieve pain (31). Hormonal therapies, including combined oral contraceptives and progestins, have demonstrated efficacy in stabilizing hormonal fluctuations and reducing both PMS and dysmenorrhea symptoms (47). Selective serotonin reuptake inhibitors (SSRIs), particularly sertraline, fluoxetine, and paroxetine, are considered the gold standard for PMDD and have demonstrated benefit in alleviating mood-related PMS symptoms (4, 45). Nutritional supplements, including calcium, vitamin D, vitamin B6, magnesium, curcumin, and omega-3 fatty acids, have varying levels of supportive evidence, though concerns persist about heterogeneity in dosing and trial quality (55, 56).

Although less frequently reported in our included studies, complementary and alternative therapies have gained increasing attention globally. Herbal remedies—including *Melissa officinalis*, anise, and *Echium amoenum*—and traditional Chinese medicine formulations have been studied with some evidence of efficacy in reducing PMS symptoms (57). Aromatherapy, progressive muscle relaxation, music medicine, and foot reflexology have also demonstrated beneficial effects in small trials, particularly in student populations (58, 59). While these interventions may be appealing to nursing students seeking non-pharmacological options, methodological variability and lack of integration into formal curricula or wellness services limit their accessibility and reliability.

A critical gap identified across the literature is the near absence of institutional interventions. Only a minority of included studies mentioned consultation with health professionals, and none described structured campus-based wellness or screening programs for menstrual health. This stands in contrast to broader recommendations that emphasize multi-level approaches, combining medical treatment, lifestyle support, and psychological interventions with institutional engagement (12, 35). Given the strong evidence for both pharmacological and non-pharmacological interventions, their underutilization among nursing students highlights both systemic neglect and cultural normalization of menstrual suffering.

### 4.6 Implications for nursing education, policy, and practice

This review highlights an urgent need to shift menstrual health from an individual responsibility to a structural priority within nursing education. Despite high prevalence rates of PMS, PMDD, and dysmenorrhea, most nursing programs worldwide lack formal wellness support, screening initiatives, or academic accommodations. This absence not only perpetuates stigma but also contributes to absenteeism and may compromise the professional development of future nurses (10, 13, 16).

#### 4.6.1 Curricular integration and student support

Nursing curricula should incorporate menstrual health education both to empower students in self-care and to prepare them as future clinicians capable of addressing menstrual disorders with empathy and evidence-based strategies (12). Routine use of validated screening tools such as the PSST and DRSP could support early identification, while flexible policies (e.g., modified deadlines, adjusted clinical hours, wellness days) would align menstrual health with other recognized accommodations for chronic conditions.

#### 4.6.2 Institutional wellness programs

Universities and nursing schools can adopt low-cost, scalable initiatives including the following:

- On-campus health and counseling services with menstrual health expertise;
- Wellness programs offering yoga, relaxation therapies, nutritional guidance, and sleep hygiene workshops; and
- Peer support or group counseling sessions to normalize help-seeking and reduce stigma.

Evidence from higher education shows that structured wellness programs including stress management workshops and yoga interventions improve student wellbeing and academic continuity (60). Tailoring such initiatives to menstrual health represents an achievable next step.

#### 4.6.3 Policy and workforce implications

Accreditation bodies and ministries of health should embed menstrual health into student wellness standards and clinical competency frameworks. Governments can play a key role by funding trials of both non-pharmacological (exercise, mindfulness, diet) and pharmacological interventions (SSRIs, oral contraceptives) for students with severe symptoms. Countries with menstrual leave policies, such as Japan and South Korea, offer potential models, though careful cultural adaptation is needed to avoid reinforcing stigma.

Nursing students represent the future health workforce. Neglecting menstrual health risks long-term consequences including burnout, absenteeism, and attrition. Institutional reforms can create a culture where menstrual health is validated as a legitimate academic and occupational concern—both modeling best practice for healthcare systems and reinforcing equity and sustainability within the profession.

### 4.7 Strengths and limitations

This review has several important strengths. First, to our knowledge, it is the most comprehensive and up-to-date systematic review and meta-analysis on PMS, PMDD, and dysmenorrhea among nursing students, covering studies published between 2016 and 2025 across diverse geographical, cultural, and educational contexts. By including 20 studies with a combined sample of over 5,000 nursing students, we provide robust prevalence estimates that underscore the substantial global burden of these conditions in this population. Second, the use of rigorous systematic review methodology, including comprehensive searches across major databases, ensures methodological credibility and replicability. Third, quality appraisal was conducted using the JBI checklist for prevalence studies, allowing for critical evaluation of study rigor. Fourth, the dual approach of quantitative synthesis (meta-analysis) and qualitative synthesis (thematic analysis) adds richness, capturing both prevalence patterns and contextual insights such as academic disruption, self-management strategies, and psychosocial burden.

Despite these strengths, some limitations must be acknowledged. High heterogeneity (*I*^2^ > 80% in some pooled estimates) was observed across studies, likely reflecting variation in diagnostic tools, definitions, cultural contexts, and reporting standards. Second, although we included only nursing students to ensure homogeneity of the study population, some studies recruited from single institutions or convenience samples, raising questions of representativeness. Third, cross-sectional designs dominated the evidence base, restricting causal inference and limiting our ability to examine trajectories over time or intervention effectiveness. Fourth, outcome measures often relied on self-reporting without clinical verification, which may introduce reporting bias. Fifth, while our review identified numerous non-pharmacological interventions (e.g., exercise, yoga, herbal supplements), very few included trials were RCTs, and most intervention studies lacked methodological rigor or long-term follow-up. Finally, although the review included studies from multiple world regions, geographic gaps remain—particularly in low- and middle-income countries—limiting the generalizability of our findings to all nursing education contexts.

The findings of the present systematic review and meta-analysis highlight the urgent need for structured institutional responses to the high burden of PMS, PMDD, and dysmenorrhea among nursing students. The evidence demonstrates not only the prevalence of menstrual disturbances but also their profound academic, psychosocial, and quality-of-life impacts. Given the dual challenges faced by nursing students—balancing demanding academic schedules with clinical responsibilities—institutions must move beyond individual coping strategies to proactive, evidence-based support systems.

### 4.8 Recommendations for policy and institutional action

The evidence presented in this review underscores a clear call to action. Menstrual disorders are not only prevalent but also profoundly disruptive to the academic and personal lives of nursing students. Institutions must therefore move beyond reliance on individual coping strategies and embed menstrual health within student wellness, curriculum design, and policy frameworks.

#### 4.8.1 Actionable recommendations include

- Routine screening and early identification should be implemented using validated tools such as the PSST or DRSP during student intake or annual health assessments.
- Integrated wellness initiatives should provide structured programs—including mindfulness, yoga, stress management workshops, and nutritional counseling—tailored to menstrual health needs.
- Accessible counseling and referral pathways should be established, with on-campus counseling services staffed by reproductive mental health expertise and clear referral links to gynecology and psychiatry.
- Curriculum integration should embed menstrual health as both a self-care topic and a professional competency for nurses.
- Equity in academic accommodations should be ensured through flexible attendance policies, adjusted clinical schedules, and wellness days for students with severe symptoms.
- Monitoring and research should be conducted to collect longitudinal data linking interventions with student performance, retention, and wellbeing, guiding continuous improvement.
- Policy advocacy should encourage nursing councils and accreditation bodies to mandate menstrual health standards, framing PMS, PMDD, and dysmenorrhea as determinants of academic success and workforce readiness.

By embedding these reforms, nursing schools and policymakers can transform menstrual health from a hidden barrier into a visible and supported component of nursing education. Such action would promote gender equity, enhance student wellbeing, and safeguard the sustainability of the nursing workforce.

## 5 Conclusion

This review underscores that menstrual disorders are not isolated individual challenges but systemic academic and public health concerns within nursing education. Despite wide heterogeneity across contexts, the consistent message is clear: PMS, PMDD, and dysmenorrhea are common, under-recognized, and undertreated, with measurable academic and social consequences. The combination of quantitative prevalence data and qualitative thematic insights calls for a shift from individual self-management to institutionally supported, evidence-based interventions. By adopting integrated wellness strategies, embedding menstrual health in curricula, and ensuring accessible care pathways, nursing schools can not only improve student wellbeing but also foster a healthier, more resilient future workforce.

## Data Availability

The original contributions presented in the study are included in the article/Supplementary Material; further inquiries can be directed to the corresponding author.
