# Correction: Prevalence of premenstrual syndrome, premenstrual dysphoric disorder, and dysmenorrhea in nursing students: a systematic review, meta-analysis, and evidence-based recommendations

**DOI:** 10.3389/fgwh.2026.1821705

**Published:** 2026-04-20

**Authors:** Sabyasachi Maity, Bharathi S. Gadad, Hansapani Rodrigo, Seham Noorani, Aneesha Usman, Chloe Lark, Mona Attarpour, Ivy Mageto, Lucas Schwartz, Anna Maria Trachuk, Dena Yaareb, Fadi Huzien, Nikhilesh Anand, Narendra Nayak, Jaime E. Mendoza, Shreya Nauhria, Samal Nauhria

**Affiliations:** 1 Department of Cellular and Integrative Physiology, Long School of Medicine, UT Health San Antonio, San Antonio, United States; 2 Department of Medical Education, UTRGV School of Medicine, Edinburg, TX, United States; 3 School of Mathematical and Statistical Sciences, UTRGV, Edinburg, TX, United States; 4 Department of Medicine, Ross University School of Medicine, Bridgetown, Barbados; 5 Department of Medicine, University of Medicine and Health Sciences, Basseterre, Saint Kitts and Nevis; 6 Department of Medicine, Avalon University School of Medicine, Willemstad, Curaçao; 7 Department of Medicine, St. George’s University School of Medicine, True Blue, Grenada; 8 Department of Microbiology, St. Matthew’s University, Georgetown, Cayman Islands; 9 PrimeWest Consortium, San Dimas Community Hospital, Graduate Medical Education, San Dimas, California, United States; 10 Child Protection, Cayman Islands Red Cross, Georgetown, Cayman Islands; 11 Civil Service College, Cayman Islands Government, Georgetown, Cayman Islands

**Keywords:** nursing students, menstrual disorders, premenstrual syndrome, dysmenorrhea, academic performance, wellbeing, premenstrual dysphoric disorder, women’s health

Error in citation

The reference for [26] was erroneously written as [Mahmoodi et al. (26)] in table 1. It should be [Mahmood KI (26)].

Reference 26 has been corrected from:

Mahmoodi M, Farajkhoda T, Nadjarzadeh A, Mahmoodabadi HZ. Online positive-oriented counseling, taking vitamin D3 tablet, online lifestyle modification training on premenstrual syndrome: a 3-armed randomized clinical trial. Sci Rep. (2023) 13(1):16631. 10.1038/s41598-023-43940-y

The correct reference should be as follows:

Mahmood KI. Premenstrual Syndrome: Presence, Knowledge, and Attitude among Female University Students. Erbil j. nurs. midwifery [Internet]. 2023 May 30 [cited 2026 Feb. 23];6(1):54–6. Available from: https://ejnm.hmu.edu.krd/index.php/ejnm/article/view/263

Error in figure/table

Wrong content

There was a mistake in figure [3] as published. [Cetin et al. (2022) events should be 72. Currently it is stated as 73]. The corrected figure 3 appears below.

**Figure 3:**
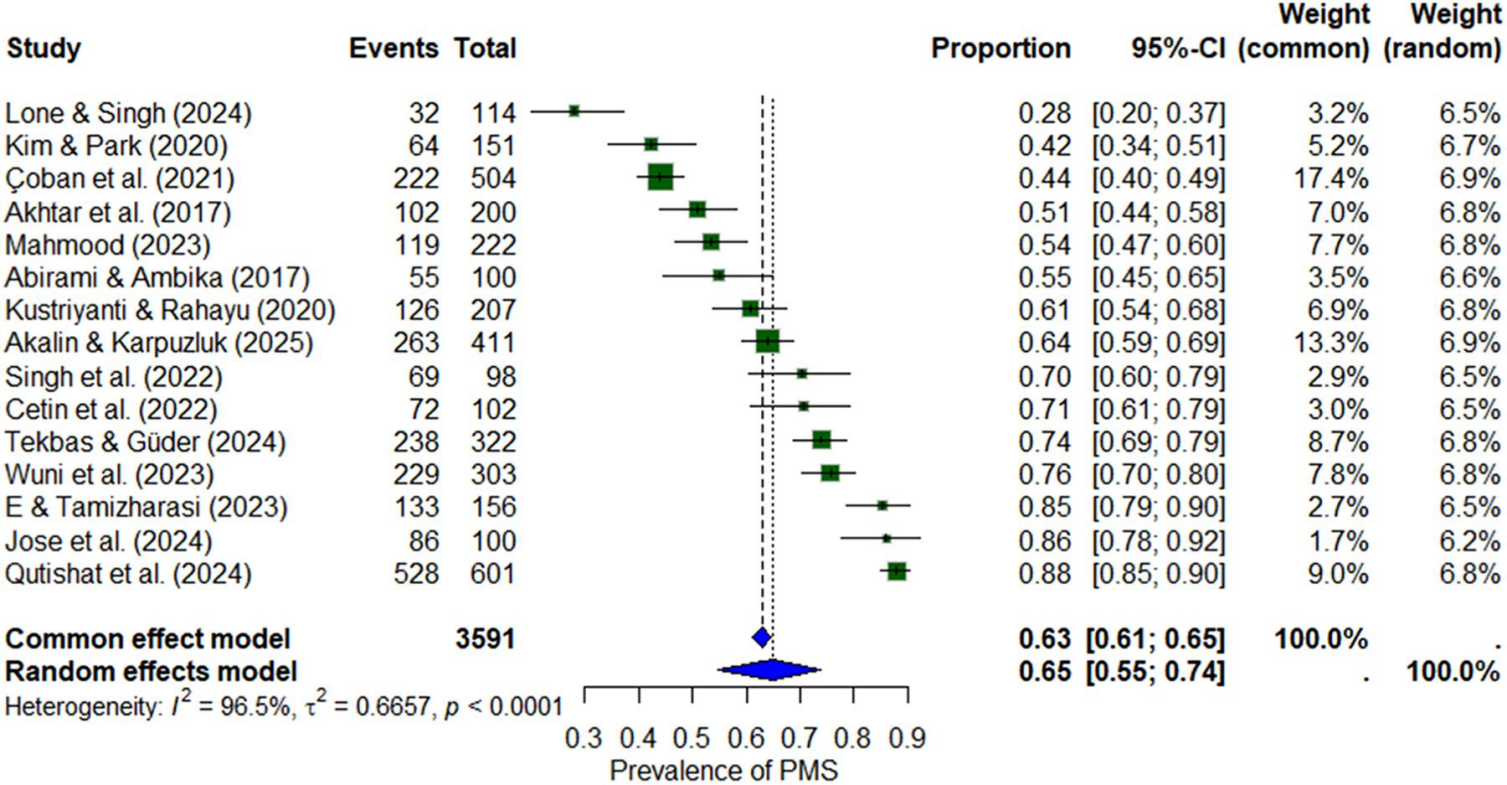
Forest plot of premenstrual syndrome (PMS) prevalence among nursing students.

This correction does not change the overall statistical significance and conclusion of the figure, and it does not change the conclusion of the paper as well. The original version of this article has been updated.

