# Supplementary material for: Prevalence of premenstrual syndrome, premenstrual dysphoric disorder, and dysmenorrhea in nursing students: a systematic review, meta-analysis, and evidence-based recommendations
Source: Front Glob Womens Health. 2026 Feb 12;6:1701704. doi: 10.3389/fgwh.2025.1701704 (PMC12935937; doi:10.3389/fgwh.2025.1701704)
Supplement: Supplementary file 1 [file Table1.docx]

**APPENDIX A: Joanna Briggs Institute (JBI) Analysis for Included Studies**

| **Author (Year)** | **Q1** | **Q2** | **Q3** | **Q4** | **Q5** | **Q6** | **Q7** | **Q8** | **Q9** | **Overall Risk of Bias** |
| --- | --- | --- | --- | --- | --- | --- | --- | --- | --- | --- |
| Akalin and Karpuzluk (2025) | Yes | Yes | Yes | Yes | Yes | Yes | Yes | Yes | Yes | Low |

**Justification for ratings:**

1. **Sample frame appropriate?**
   Yes – All female nursing students at a university, matching the target population.
2. **Sampling method appropriate?**
   Yes – Clear inclusion/exclusion; all eligible students recruited.
3. **Sample size adequate?**
   Yes – Large sample (n=411) for prevalence, well above minimum thresholds.
4. **Subjects/setting described in detail?**
   Yes – Clear description of institution, period, demographics, inclusion/exclusion.
5. **Analysis coverage adequate?**
   Yes – Nearly all recruited students included; minimal exclusion/loss.
6. **Valid identification methods?**
   Yes – PMSS and DASS, validated tools, described and cited.
7. **Standard, reliable measurement?**
   Yes – Same scales/questionnaires for all participants.
8. **Appropriate statistical analysis?**
   Yes – Statistical methods described and appropriate (t-tests, ANOVA, correlations).
9. **Adequate response rate/management?**
   Yes – High response rate reported (all eligible included or >90%).

**Overall:**

- Low risk of bias for prevalence estimation.

| **Author (Year)** | **Q1** | **Q2** | **Q3** | **Q4** | **Q5** | **Q6** | **Q7** | **Q8** | **Q9** | **Overall Risk of Bias** |
| --- | --- | --- | --- | --- | --- | --- | --- | --- | --- | --- |
| Tekbaş and Güder (2024) | Yes | Yes | Yes | Yes | Yes | Yes | Yes | Yes | Yes | Low |

**Justification for ratings:**

1. **Sample frame appropriate?**
   Yes – All female nursing students at a university; clearly defined.
2. **Sampling method appropriate?**
   Yes – All eligible students approached; voluntary participation.
3. **Sample size adequate?**
   Yes – Sample of 322 out of 350 students (92% participation); strong for prevalence.
4. **Subjects/setting described in detail?**
   Yes – Clear information on population, institution, data collection, inclusion/exclusion.
5. **Analysis coverage adequate?**
   Yes – Nearly entire eligible cohort included, minimal exclusion.
6. **Valid identification methods?**
   Yes – PMSS (validated scale), methods described.
7. **Standard, reliable measurement?**
   Yes – Same scale and data collection for all participants.
8. **Appropriate statistical analysis?**
   Yes – Descriptive, t-test, ANOVA, post-hoc, correlations; methods appropriate.
9. **Adequate response rate/management?**
   Yes – Very high response rate (92%).

**Overall:**

- Low risk of bias for prevalence estimation.

| **Author (Year)** | **Q1** | **Q2** | **Q3** | **Q4** | **Q5** | **Q6** | **Q7** | **Q8** | **Q9** | **Overall Risk of Bias** |
| --- | --- | --- | --- | --- | --- | --- | --- | --- | --- | --- |
| Qutishat et al. (2024) | Yes | Yes | Yes | Yes | Yes | Yes | Yes | Yes | Yes | Low |

**Justification for ratings:**

1. **Sample frame appropriate?**
   Yes – Included female undergraduate college students in Oman; frame matches target population.
2. **Sampling method appropriate?**
   Yes – Convenience sampling, but covered large portion of accessible population; justified and typical for prevalence studies.
3. **Sample size adequate?**
   Yes – Large sample (n=601) for prevalence estimate.
4. **Subjects/setting described in detail?**
   Yes – Age, setting, and population clearly described.
5. **Analysis coverage adequate?**
   Yes – All completed questionnaires included.
6. **Valid identification methods?**
   Yes – Used validated PMSS and attitude scales; reliability reported.
7. **Standard, reliable measurement?**
   Yes – Same measurement tools applied for all.
8. **Appropriate statistical analysis?**
   Yes – Descriptive statistics, regression; appropriate and clearly described.
9. **Adequate response rate/management?**
   Yes – High completion rate (reported 601/unknown approached, but no evidence of major non-response bias).

**Overall:**

- Low risk of bias.

| **Author (Year)** | **Q1** | **Q2** | **Q3** | **Q4** | **Q5** | **Q6** | **Q7** | **Q8** | **Q9** | **Overall Risk of Bias** |
| --- | --- | --- | --- | --- | --- | --- | --- | --- | --- | --- |
| Lone and Singh (2024) | Yes | Yes | Yes | Yes | Yes | Yes | Yes | Yes | Yes | Low |

**Justification for ratings:**

1. **Sample frame appropriate?**
   Yes – All nursing students at a specific nursing college; fits target.
2. **Sampling method appropriate?**
   Yes – All eligible students were approached and recruited.
3. **Sample size adequate?**
   Yes – n=148; reasonable for prevalence of PMDD/PMS in this context.
4. **Subjects/setting described in detail?**
   Yes – Detailed description of participants and institutional context.
5. **Analysis coverage adequate?**
   Yes – All eligible students who consented were included in analysis.
6. **Valid identification methods?**
   Yes – Used the PSST, a validated screening tool.
7. **Standard, reliable measurement?**
   Yes – All students assessed with same tool.
8. **Appropriate statistical analysis?**
   Yes – Descriptive stats; methods appropriate and described.
9. **Adequate response rate/management?**
   Yes – High response rate; minimal loss/exclusion.

**Overall:**

- Low risk of bias.

| **Author (Year)** | **Q1** | **Q2** | **Q3** | **Q4** | **Q5** | **Q6** | **Q7** | **Q8** | **Q9** | **Overall Risk of Bias** |
| --- | --- | --- | --- | --- | --- | --- | --- | --- | --- | --- |
| Jose et al. (2024) | Yes | Yes | Yes | Yes | Yes | Yes | Yes | Yes | Yes | Low |

**Justification for ratings:**

1. **Sample frame appropriate?**
   Yes – Targeted late adolescent female nursing students at a private college.
2. **Sampling method appropriate?**
   Yes – Random sampling used for main survey; focus group from those screened positive.
3. **Sample size adequate?**
   Yes – n=100 (quantitative), n=10 (qualitative focus group); adequate for both arms.
4. **Subjects/setting described in detail?**
   Yes – Demographics, institutional context, and sampling clearly described.
5. **Analysis coverage adequate?**
   Yes – All eligible and consenting students included.
6. **Valid identification methods?**
   Yes – Used standardized PMS diagnostic criteria (Stainer & Wilkin).
7. **Standard, reliable measurement?**
   Yes – All participants assessed with same tool.
8. **Appropriate statistical analysis?**
   Yes – Descriptive, thematic analysis for qualitative, appropriate for mixed-methods.
9. **Adequate response rate/management?**
   Yes – High response/completion rate.

**Overall:**

- Low risk of bias.

| **Author (Year)** | **Q1** | **Q2** | **Q3** | **Q4** | **Q5** | **Q6** | **Q7** | **Q8** | **Q9** | **Overall Risk of Bias** |
| --- | --- | --- | --- | --- | --- | --- | --- | --- | --- | --- |
| Wuni et al. (2023) | Yes | Yes | Yes | Yes | Yes | Yes* | Yes | Yes | Yes | Low |

**Justification for ratings:**

1. **Sample frame appropriate?**
   *Yes* – All nurse and midwife trainees at three Northern Ghana colleges; representative.
2. **Sampling method appropriate?**
   *Yes* – Proportionate stratified random sampling used.
3. **Sample size adequate?**
   *Yes* – n=303; sample size calculation described; large enough for prevalence.
4. **Subjects/setting described in detail?**
   *Yes* – Details of colleges, students, sampling, and setting provided.
5. **Analysis coverage adequate?**
   *Yes* – High coverage; all eligible and consenting students included.
6. **Valid identification methods?**
   *Yes* – Structured questionnaire and VAS; VAS validated, but structured questionnaire was piloted (not externally validated); *still acceptable for context*.
7. **Standard, reliable measurement?**
   *Yes* – All students assessed using the same instrument/procedures.
8. **Appropriate statistical analysis?**
   *Yes* – Descriptive statistics and logistic regression; methods clear and appropriate.
9. **Adequate response rate/management?**
   *Yes* – High response rate.

**Overall:**

- *Low risk of bias.* (Note: Validated tool for pain, but the questionnaire was not externally validated—common in LMIC studies.)

| **Author (Year)** | **Q1** | **Q2** | **Q3** | **Q4** | **Q5** | **Q6** | **Q7** | **Q8** | **Q9** | **Overall Risk of Bias** |
| --- | --- | --- | --- | --- | --- | --- | --- | --- | --- | --- |
| Mahmood (2023) | Yes | Yes | Yes | Yes | Yes | Yes* | Yes | Yes | Yes | Low–Moderate |

**Justification for ratings:**

1. **Sample frame appropriate?**
   Yes – Targeted all female university students at a College of Nursing.
2. **Sampling method appropriate?**
   Yes – All eligible students could participate in the online survey.
3. **Sample size adequate?**
   Yes – n=222; appropriate for prevalence.
4. **Subjects/setting described in detail?**
   Yes – Student characteristics, inclusion/exclusion, setting described.
5. **Analysis coverage adequate?**
   Yes – All valid responses analyzed.
6. **Valid identification methods?**
   Yes (with caution) – Used ACOG criteria for PMS (validated), but custom scales for knowledge/attitude/impact (piloted but not externally validated).
7. **Standard, reliable measurement?**
   Yes – All students used the same survey tools and online format.
8. **Appropriate statistical analysis?**
   Yes – Descriptive stats, appropriate tests used.
9. **Adequate response rate/management?**
   Yes – Response rate is acceptable for online surveys; addressed missing data.

**Overall:**

- Low to moderate risk of bias (due to custom non-validated knowledge/attitude subscales, but main PMS outcome based on validated criteria).

| **Author (Year)** | **Q1** | **Q2** | **Q3** | **Q4** | **Q5** | **Q6** | **Q7** | **Q8** | **Q9** | **Overall Risk of Bias** |
| --- | --- | --- | --- | --- | --- | --- | --- | --- | --- | --- |
| E and Tamizharasi (2023) | Yes | Yes | Yes | Yes | Yes | No | Yes | Yes | Yes | Moderate |

**Justification for ratings:**

1. **Sample frame appropriate?**
   Yes – Adolescent girls at a specific institution; aligns with target.
2. **Sampling method appropriate?**
   Yes – Non-probability convenience sampling, typical for setting.
3. **Sample size adequate?**
   Yes – n=156; suitable for prevalence estimate.
4. **Subjects/setting described in detail?**
   Yes – Details on participants and setting included.
5. **Analysis coverage adequate?**
   Yes – All participants included.
6. **Valid identification methods?**
   No – Used a custom, researcher-developed checklist/interview tool for PMS, not externally validated.
7. **Standard, reliable measurement?**
   Yes – Same tool used for all.
8. **Appropriate statistical analysis?**
   Yes – Appropriate statistical methods (descriptive, associations).
9. **Adequate response rate/management?**
   Yes – High response rate.

**Overall:**

- Moderate risk of bias (due to lack of validation for PMS measurement tool).

| **Author (Year)** | **Q1** | **Q2** | **Q3** | **Q4** | **Q5** | **Q6** | **Q7** | **Q8** | **Q9** | **Overall Risk of Bias** |
| --- | --- | --- | --- | --- | --- | --- | --- | --- | --- | --- |
| Singh et al. (2022) | Yes | Yes | Yes | Yes | Yes | No | Yes | Yes | Yes | Moderate |

**Justification for ratings:**

1. **Sample frame appropriate?**
   Yes – Targeted all female nursing and medical undergraduates at a tertiary institute.
2. **Sampling method appropriate?**
   Yes – Voluntary response, but all eligible approached; typical for cross-sectional studies.
3. **Sample size adequate?**
   Yes – n=188 nursing students (from a larger pool); suitable for prevalence estimation.
4. **Subjects/setting described in detail?**
   Yes – Details on demographics, inclusion/exclusion, and institutional context provided.
5. **Analysis coverage adequate?**
   Yes – All valid responses included in analysis.
6. **Valid identification methods?**
   No – Used a custom, semi-structured questionnaire not externally validated.
7. **Standard, reliable measurement?**
   Yes – All participants completed the same instrument.
8. **Appropriate statistical analysis?**
   Yes – Appropriate (descriptive, associations); analysis clearly described.
9. **Adequate response rate/management?**
   Yes – Response rate and missing data addressed.

**Overall:**

- Moderate risk of bias (due to use of non-validated measurement tool for menstrual disorders).

| **Author (Year)** | **Q1** | **Q2** | **Q3** | **Q4** | **Q5** | **Q6** | **Q7** | **Q8** | **Q9** | **Overall Risk of Bias** |
| --- | --- | --- | --- | --- | --- | --- | --- | --- | --- | --- |
| Çoban et al. (2021) | Yes | Yes | Yes | Yes | Yes | Yes | Yes | Yes | Yes | Low |

**Justification for ratings:**

1. **Sample frame appropriate?**
   Yes – Female nursing students at a single university; representative.
2. **Sampling method appropriate?**
   Yes – All eligible students approached.
3. **Sample size adequate?**
   Yes – n=504; very robust sample for prevalence.
4. **Subjects/setting described in detail?**
   Yes – Age, gender, year, university described.
5. **Analysis coverage adequate?**
   Yes – High participation, all data included.
6. **Valid identification methods?**
   Yes – PSST, EAT-26, and TFEQ-R18, all internationally validated tools.
7. **Standard, reliable measurement?**
   Yes – Same tools administered to all participants.
8. **Appropriate statistical analysis?**
   Yes – Descriptive statistics and appropriate tests for associations.
9. **Adequate response rate/management?**
   Yes – High response rate and no indication of significant bias.

**Overall:**

- Low risk of bias.

| **Author (Year)** | **Q1** | **Q2** | **Q3** | **Q4** | **Q5** | **Q6** | **Q7** | **Q8** | **Q9** | **Overall Risk of Bias** |
| --- | --- | --- | --- | --- | --- | --- | --- | --- | --- | --- |
| Kim and Park (2020) | Yes | Yes | Yes | Yes | Yes | Yes | Yes | Yes | Yes | Low |

**Justification for ratings:**

1. **Sample frame appropriate?**
   Yes – Female nursing students at a university; matches target population.
2. **Sampling method appropriate?**
   Yes – All eligible students invited; voluntary participation.
3. **Sample size adequate?**
   Yes – n=151 (retrospective), n=17 (prospective); sample adequate for prevalence and validation comparison.
4. **Subjects/setting described in detail?**
   Yes – Demographics, inclusion/exclusion, data collection period, and setting described.
5. **Analysis coverage adequate?**
   Yes – Nearly all eligible included; clear on drop-outs.
6. **Valid identification methods?**
   Yes – DRSP and PBAC, both validated and widely used.
7. **Standard, reliable measurement?**
   Yes – All participants assessed with same validated tools.
8. **Appropriate statistical analysis?**
   Yes – Descriptive, comparison, and validation stats used appropriately.
9. **Adequate response rate/management?**
   Yes – High participation and management of missing data.

**Overall:**

- Low risk of bias.

| **Author (Year)** | **Q1** | **Q2** | **Q3** | **Q4** | **Q5** | **Q6** | **Q7** | **Q8** | **Q9** | **Overall Risk of Bias** |
| --- | --- | --- | --- | --- | --- | --- | --- | --- | --- | --- |
| Kustriyanti and Rahayu (2020) | Yes | Yes | Yes | Yes | Yes | Yes | Yes | Yes | Yes | Low |

**Justification for ratings:**

1. **Sample frame appropriate?**
   *Yes* – Female nursing students at a health sciences college; matches target group.
2. **Sampling method appropriate?**
   *Yes* – All eligible students approached; voluntary, but typical for prevalence.
3. **Sample size adequate?**
   *Yes* – n=207; adequate for prevalence and QOL estimation.
4. **Subjects/setting described in detail?**
   *Yes* – Demographics, institutional setting, inclusion criteria detailed.
5. **Analysis coverage adequate?**
   *Yes* – All included, exclusions described.
6. **Valid identification methods?**
   *Yes* – Used validated international tools: ACOG PMS, DSM-IV PMDD, WHOQOL-BREF.
7. **Standard, reliable measurement?**
   *Yes* – Standardized questionnaires administered to all.
8. **Appropriate statistical analysis?**
   *Yes* – Descriptive and comparative stats appropriate for aims.
9. **Adequate response rate/management?**
   *Yes* – High participation; response rate and data completeness addressed.

**Overall:**

- *Low risk of bias.*

| **Author (Year)** | **Q1** | **Q2** | **Q3** | **Q4** | **Q5** | **Q6** | **Q7** | **Q8** | **Q9** | **Overall Risk of Bias** |
| --- | --- | --- | --- | --- | --- | --- | --- | --- | --- | --- |
| Abreu-Sánchez et al. (2020) | Yes | Yes | Yes | Yes | Yes | Yes* | Yes | Yes | Yes | Low–Moderate |

**Justification for ratings:**

1. **Sample frame appropriate?**
   Yes – Female nursing students at a university; matches intended target.
2. **Sampling method appropriate?**
   Yes – Voluntary participation, but all eligible students invited.
3. **Sample size adequate?**
   Yes – n=258; sufficient for prevalence.
4. **Subjects/setting described in detail?**
   Yes – Demographics, institutional setting, period, criteria described.
5. **Analysis coverage adequate?**
   Yes – All valid responses included in analysis.
6. **Valid identification methods?**
   Yes (with caution) – Used a self-report ad hoc symptom checklist (not externally validated), but VAS for pain is validated and widely used.
7. **Standard, reliable measurement?**
   Yes – All completed the same questionnaire and scales.
8. **Appropriate statistical analysis?**
   Yes – Descriptive and logistic regression, clearly described.
9. **Adequate response rate/management?**
   Yes – High response rate and participation.

**Overall:**

- Low–moderate risk of bias (primarily due to use of a non-validated symptom checklist, but otherwise rigorous).

| **Author (Year)** | **Q1** | **Q2** | **Q3** | **Q4** | **Q5** | **Q6** | **Q7** | **Q8** | **Q9** | **Overall Risk of Bias** |
| --- | --- | --- | --- | --- | --- | --- | --- | --- | --- | --- |
| Vlachou et al. (2019) | Yes | Yes | Yes | Yes | Yes | Yes* | Yes | Yes | Yes | Low–Moderate |

**Justification for ratings:**

1. **Sample frame appropriate?**
   Yes – Female nursing students at a Greek university.
2. **Sampling method appropriate?**
   Yes – All eligible students invited to participate; cross-sectional.
3. **Sample size adequate?**
   Yes – n=637; very robust for prevalence study.
4. **Subjects/setting described in detail?**
   Yes – Demographics, setting, inclusion/exclusion described.
5. **Analysis coverage adequate?**
   Yes – Nearly all eligible included in analysis.
6. **Valid identification methods?**
   Yes (with caution) – Used custom questionnaire for dysmenorrhea (piloted, not externally validated), but VAS for pain is validated.
7. **Standard, reliable measurement?**
   Yes – All completed same instruments.
8. **Appropriate statistical analysis?**
   Yes – Appropriate methods, including logistic regression.
9. **Adequate response rate/management?**
   Yes – High response rate reported.

**Overall:**

- Low–moderate risk of bias (due to use of a piloted but non-validated symptom questionnaire).

| **Author (Year)** | **Q1** | **Q2** | **Q3** | **Q4** | **Q5** | **Q6** | **Q7** | **Q8** | **Q9** | **Overall Risk of Bias** |
| --- | --- | --- | --- | --- | --- | --- | --- | --- | --- | --- |
| Fernández-Martínez et al. (2018) | Yes | Yes | Yes | Yes | Yes | Yes* | Yes | Yes | Yes | Low–Moderate |

**Justification for ratings:**

1. **Sample frame appropriate?**
   Yes – Female nursing students at a Spanish university.
2. **Sampling method appropriate?**
   Yes – All eligible students invited; voluntary participation.
3. **Sample size adequate?**
   Yes – n=258; sufficient for prevalence.
4. **Subjects/setting described in detail?**
   Yes – Demographics, setting, period, criteria described.
5. **Analysis coverage adequate?**
   Yes – All included, high coverage.
6. **Valid identification methods?**
   Yes (with caution) – Used custom questionnaire for dysmenorrhea (piloted but not externally validated); VAS for pain is validated.
7. **Standard, reliable measurement?**
   Yes – All participants completed same instruments.
8. **Appropriate statistical analysis?**
   Yes – Descriptive, bivariate, logistic regression; methods described.
9. **Adequate response rate/management?**
   Yes – High response rate.

**Overall:**

- Low–moderate risk of bias (due to non-validated symptom questionnaire, but other aspects robust).

| **Author (Year)** | **Q1** | **Q2** | **Q3** | **Q4** | **Q5** | **Q6** | **Q7** | **Q8** | **Q9** | **Overall Risk of Bias** |
| --- | --- | --- | --- | --- | --- | --- | --- | --- | --- | --- |
| Akhtar et al. (2017) | Yes | Yes | Yes | Yes | Yes | Yes | Yes | Yes | Yes | Low |

**Justification for ratings:**

1. **Sample frame appropriate?**
   Yes – Female nursing students at a tertiary care hospital.
2. **Sampling method appropriate?**
   Yes – All eligible students approached and included.
3. **Sample size adequate?**
   Yes – n=200; appropriate for prevalence.
4. **Subjects/setting described in detail?**
   Yes – Participant demographics, hospital setting, and period provided.
5. **Analysis coverage adequate?**
   Yes – All participants included in analysis.
6. **Valid identification methods?**
   Yes – Used the Revised Premenstrual Tension Syndrome Scale (Observer Rating), described as validated.
7. **Standard, reliable measurement?**
   Yes – All students assessed using the same instrument.
8. **Appropriate statistical analysis?**
   Yes – Descriptive statistics, methods clear.
9. **Adequate response rate/management?**
   Yes – High response and completion rate.

**Overall:**

- Low risk of bias.

| **Author (Year)** | **Q1** | **Q2** | **Q3** | **Q4** | **Q5** | **Q6** | **Q7** | **Q8** | **Q9** | **Overall Risk of Bias** |
| --- | --- | --- | --- | --- | --- | --- | --- | --- | --- | --- |
| Abirami and Ambika (2017) | Yes | Yes | Yes | Yes | Yes | No | Yes | Yes | Yes | Moderate |

**Justification for ratings:**

1. **Sample frame appropriate?**
   Yes – Adolescent nursing students at a university; appropriate for target.
2. **Sampling method appropriate?**
   Yes – Non-probability convenience sampling; all eligible included.
3. **Sample size adequate?**
   Yes – n=100; sufficient for descriptive prevalence study.
4. **Subjects/setting described in detail?**
   Yes – Age, setting, and eligibility criteria described.
5. **Analysis coverage adequate?**
   Yes – All included, no exclusions reported.
6. **Valid identification methods?**
   No – Used a researcher-developed questionnaire for PMS, not externally validated.
7. **Standard, reliable measurement?**
   Yes – Same questionnaire given to all participants.
8. **Appropriate statistical analysis?**
   Yes – Descriptive and inferential statistics used appropriately.
9. **Adequate response rate/management?**
   Yes – All eligible participated.

**Overall:**

- Moderate risk of bias (due to non-validated measurement tool for PMS).

| **Author (Year)** | **Q1** | **Q2** | **Q3** | **Q4** | **Q5** | **Q6** | **Q7** | **Q8** | **Q9** | **Overall Risk of Bias** |
| --- | --- | --- | --- | --- | --- | --- | --- | --- | --- | --- |
| Cetin et al. (2022) | Yes | Yes | Yes | Yes | Yes | Yes | Yes | Yes | Yes | Low |

**Justification:**

- Target population, appropriate sampling and size, detailed setting, full coverage of sample, validated tool (PMSS), standard measurement, appropriate statistics, high response rate.
- PMSS tool had very high internal consistency (α=.97) in this study.

| **Author (Year)** | **Q1** | **Q2** | **Q3** | **Q4** | **Q5** | **Q6** | **Q7** | **Q8** | **Q9** | **Overall Risk of Bias** |
| --- | --- | --- | --- | --- | --- | --- | --- | --- | --- | --- |
| Osman & El-Houfey (2016) | Yes | Yes | Yes | Yes | Yes | Yes* | Yes | Yes | Yes | Low–moderate |

**Justification:**

- Sample frame, sampling, and size appropriate; all participants described and included.
- Used a self-developed questionnaire and a validated scoring system (with pilot and expert review, α=0.95).
- Interventions and activities detailed, and appropriate statistical analysis used.
- Most risk is due to the use of some self-developed questions and lack of external validation for all scales, but overall methods robust.

| **Author (Year)** | **Q1** | **Q2** | **Q3** | **Q4** | **Q5** | **Q6** | **Q7** | **Q8** | **Q9** | **Overall Risk of Bias** |
| --- | --- | --- | --- | --- | --- | --- | --- | --- | --- | --- |
| Shewte and Sirpurkar (2016) | Yes | Yes | Yes | Yes | Yes | Yes* | Yes | Yes | Yes | Low–moderate |

**Justification:**

- Sample frame and size appropriate; setting and subjects described.
- Standard and validated HRQoL (SF-36) used, but diagnosis of dysmenorrhea based on self-report/custom definitions (as in many studies).
- Interventions and academic/social impacts well described.
- Appropriate statistics.
- Minor risk from self-developed symptom items and potential recall bias, but otherwise strong.
