# Supplementary material for: Prevalence of premenstrual syndrome, premenstrual dysphoric disorder, and dysmenorrhea in nursing students: a systematic review, meta-analysis, and evidence-based recommendations
Source: Front Glob Womens Health. 2026 Feb 12;6:1701704. doi: 10.3389/fgwh.2025.1701704 (PMC12935937; doi:10.3389/fgwh.2025.1701704)

**Funnel plots for each outcome (dysmenorrhea, PMS, PMDD and Combined PMDD/Severe PMS) are provided in the Supplementary Materials (Fig. S1-S4).**


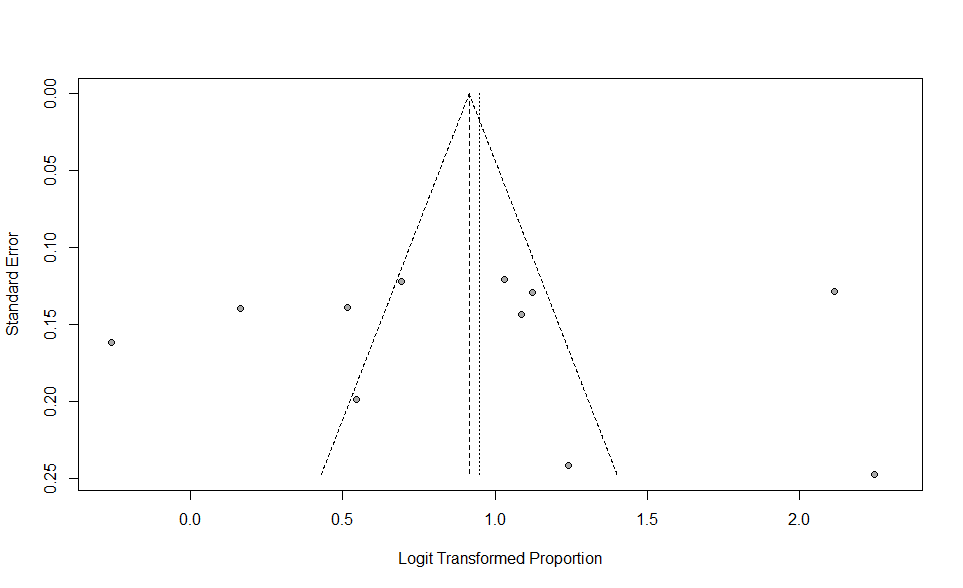


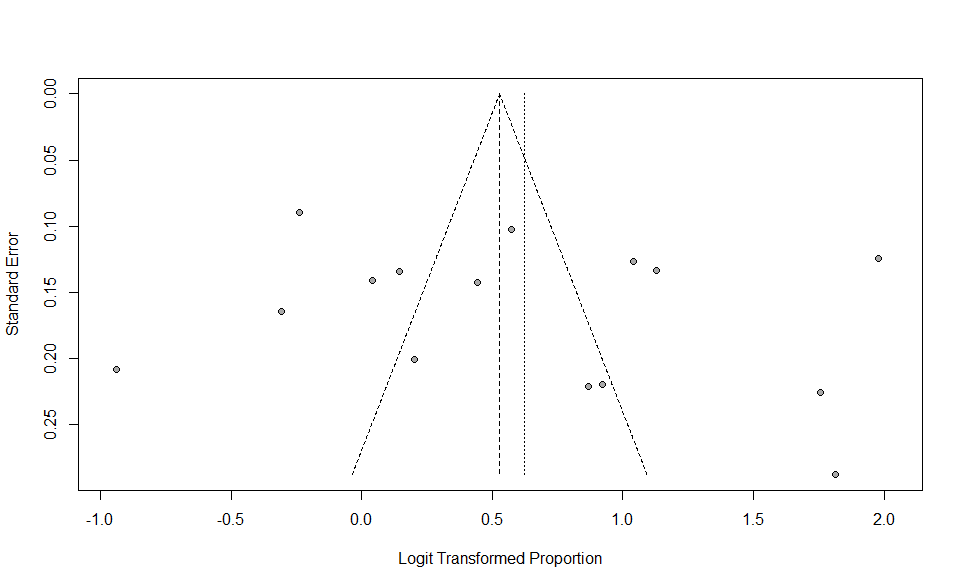


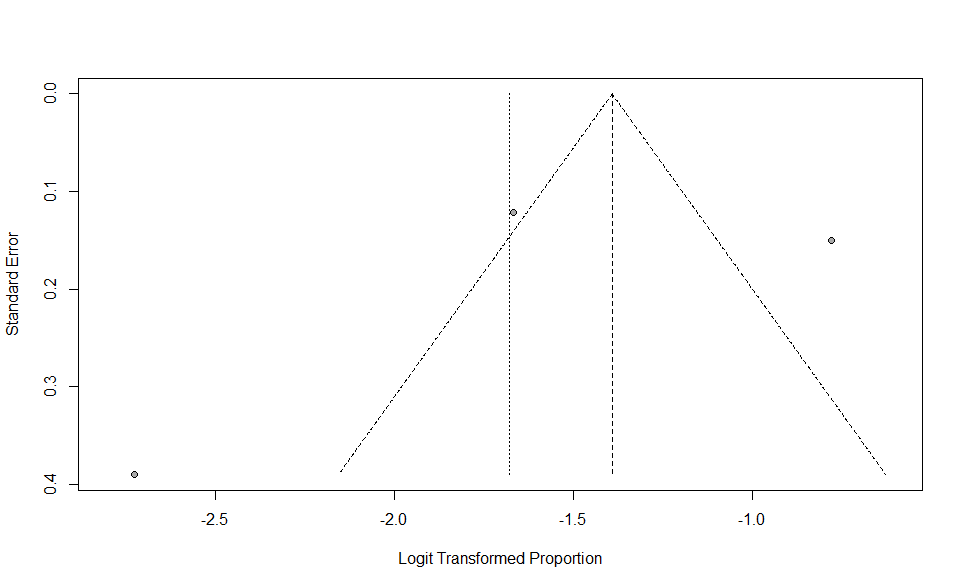


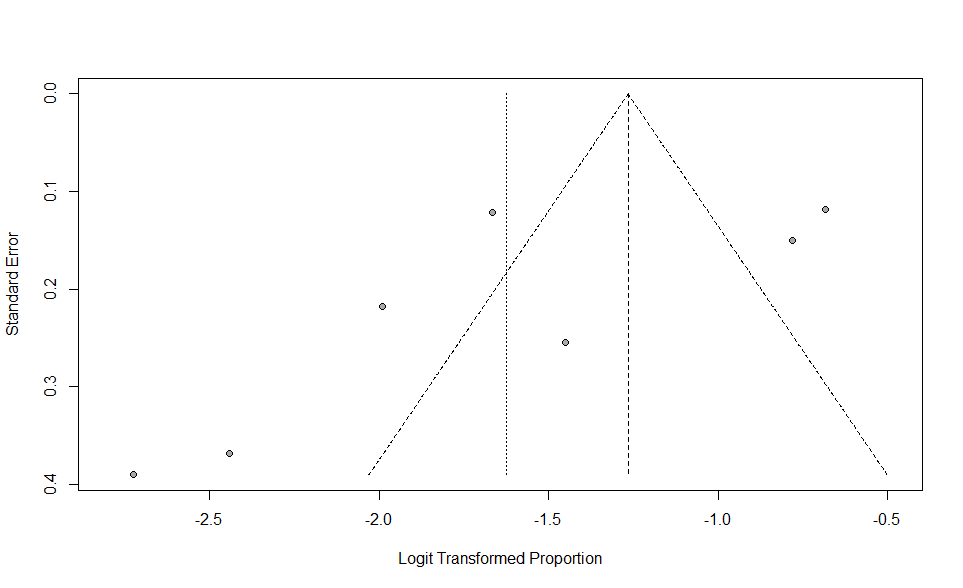

Supplement: Supplementary file 2 [file Table2.docx]
